# Supplementary material for: Ileal Crohn’s Disease Exhibits Reduced Activity of Phospholipase C-β3-Dependent Wnt/β-Catenin Signaling Pathway
Source: Cells. 2024 Jun 5;13(11):986. doi: 10.3390/cells13110986 (PMC11171731; doi:10.3390/cells13110986)
Supplement: Supplementary file 1 [file cells-13-00986-s001.zip › cells-3021175-supplementary.pdf]

**Supplemental Information**

# Ileal Crohn's disease exhibits reduced activity of phospholipase C-β3-dependent Wnt/β-catenin signaling pathway

Tomoaki Ando[1,2,11], Ikuo Takazawa[1,3,11], Zach Spencer[4], Ryoji Ito[1,5],
Yoshiaki Tomimori[1,3], Zbigniew Mikulski[6], Kenji Matsumoto[7], Tohru Ishitani[8],
Lee A. Denson[9,10], Yu Kawakami[1], Yuko Kawakami[1], Jiro Kitaura[2], Yashi Ahmed[4],
and Toshiaki Kawakami[1]*

Content:

Table S1 & S2
Figures S1-S9
Uncropped images of western blots
Videos 1-6

Table S1. Mouse genes upregulated in ileum of *Plcb3*$^{-/-}$ mice

| Gene symbol | Gene name | Fold change (KO vs WT) | q-value |
|---|---|---|---|
| Retnlb | resistin like beta | 375.5 | 5.0E-05 |
| Pla2g4c | phospholipase A2, group IVC (cytosolic, calcium-independent) | 199.0 | 1.6E-04 |
| U90926 | cDNA sequence U90926 | 67.2 | 1.1E-04 |
| Duoxa2 | dual oxidase maturation factor 2 | 36.1 | 8.0E-05 |
| Gsdmcl-ps | gasdermin C-like, pseudogene | 29.8 | 1.1E-05 |
| Defb1 | defensin beta 1 | 27.2 | 1.0E-04 |
| Krt84 | keratin 84 | 25.9 | 2.4E-05 |
| Spna1 | spectrin alpha 1 | 24.9 | 1.2E-05 |
| Gml | GPI anchored molecule like protein | 22.4 | 1.8E-04 |
| 4930597O21Rik | RIKEN cDNA 4930597O21 gene | 22.2 | 3.6E-04 |
| Pnliprp1 | pancreatic lipase related protein 1 | 17.9 | 1.1E-04 |
| Fut2 | fucosyltransferase 2 | 17.6 | 7.4E-05 |
| Ccl24 | chemokine (C-C motif) ligand 24 | 16.5 | 6.4E-05 |
| Gsdmc3 | gasdermin C3 | 16.1 | 5.8E-05 |
| Saa2 | serum amyloid A 2 | 15.9 | 2.1E-03 |
| Gsdmc | gasdermin C | 15.1 | 4.7E-05 |
| Capn13 | calpain 13 | 14.7 | 4.6E-05 |
| Il1rl1 | interleukin 1 receptor-like 1 | 14.6 | 2.0E-04 |
| Saa1 | serum amyloid A 1 | 14.0 | 4.0E-03 |
| Gsdmc4 | gasdermin C4 | 13.6 | 4.6E-05 |
| Duox2 | dual oxidase 2 | 13.6 | 1.4E-04 |
| Islr2 | immunoglobulin superfamily containing leucine-rich repeat 2 | 13.3 | 2.8E-04 |
| Pnliprp2 | pancreatic lipase-related protein 2 | 12.9 | 3.1E-05 |
| S100a9 | S100 calcium binding protein A9 (calgranulin B) | 12.4 | 1.8E-03 |
| Nfx1 | nuclear transcription factor, X-box binding 1 | 12.0 | 2.8E-05 |
| 1810030J14Rik | RIKEN cDNA 1810030J14 gene | 11.1 | 1.7E-03 |
| Mbl2 | mannose-binding lectin (protein C) 2 | 10.8 | 2.8E-04 |
| Abpe | androgen binding protein epsilon | 9.9 | 4.6E-05 |
| Retnlg | resistin like gamma | 9.7 | 7.5E-05 |
| Cxcl5 | chemokine (C-X-C motif) ligand 5 | 9.4 | 3.1E-05 |
| Abpd | androgen binding protein delta | 9.2 | 5.0E-05 |
| Gm6504 | predicted gene 6504 | 9.0 | 5.7E-05 |
| Me1 | malic enzyme 1, NADP(+)-dependent, cytosolic | 8.9 | 5.0E-05 |
| Cebpe | CCAAT/enhancer binding protein (C/EBP), epsilon | 8.2 | 1.3E-04 |
| Cfi | complement component factor i | 8.2 | 4.9E-04 |
| Kcnj16 | potassium inwardly-rectifying channel, subfamily J, member 16 | 7.6 | 6.1E-05 |
| AA467197 | expressed sequence AA467197 | 7.2 | 2.5E-04 |
| Clps | colipase, pancreatic | 7.1 | 2.3E-04 |
| Ly6k | lymphocyte antigen 6 complex, locus K | 6.6 | 2.8E-03 |
| Car4 | carbonic anhydrase 4 | 6.6 | 1.1E-04 |
| Apoh | apolipoprotein H | 6.3 | 4.2E-04 |
| Reg4 | regenerating islet-derived family, member 4 | 6.2 | 1.0E-04 |
| Il13 | interleukin 13 | 6.0 | 5.8E-05 |

| | | | |
|---|---|---|---|
| Daf2 | decay accelerating factor 2 | 5.8 | 1.1E-04 |
| Ang3 | angiogenin, ribonuclease A family, member 3 | 5.8 | 9.8E-04 |
| Gm9223 | predicted gene 9223 | 5.7 | 9.3E-05 |
| Ang2 | angiogenin, ribonuclease A family, member 2 | 5.5 | 9.6E-04 |
| 2010001M09Rik | RIKEN cDNA 2010001M09 gene | 5.5 | 5.8E-05 |
| Pla2g5 | phospholipase A2, group V | 5.4 | 9.6E-05 |
| Cd55 | CD55 antigen | 5.3 | 7.4E-05 |
| S100a8 | S100 calcium binding protein A8 (calgranulin A) | 5.2 | 1.6E-03 |
| Ceacam12 | carcinoembryonic antigen-related cell adhesion molecule 12 | 5.2 | 1.8E-04 |
| Neto1 | neuropilin (NRP) and tolloid (TLL)-like 1 | 5.2 | 6.6E-05 |
| Adm | adrenomedullin | 5.1 | 1.1E-04 |
| Pla2g2e | phospholipase A2, group IIE | 5.0 | 6.4E-05 |
| Gpnmb | glycoprotein (transmembrane) nmb | 4.9 | 3.0E-04 |
| LOC100505358 | uncharacterized LOC100505358 | 4.9 | 3.7E-04 |
| Hemt1 | hematopoietic cell transcript 1 | 4.9 | 4.9E-03 |
| Alox5 | arachidonate 5-lipoxygenase | 4.8 | 2.8E-04 |
| Ly6d | lymphocyte antigen 6 complex, locus D | 4.6 | 5.4E-04 |
| Duoxa1 | dual oxidase maturation factor 1 | 4.5 | 4.2E-04 |
| Rab44 | RAB44, member RAS oncogene family | 4.5 | 8.0E-05 |
| Expi | extracellular proteinase inhibitor | 4.5 | 5.7E-04 |
| Ear11 | eosinophil-associated, ribonuclease A family, member 11 | 4.5 | 8.5E-05 |
| Csn3 | casein kappa | 4.4 | 1.1E-04 |
| Ccl8 | chemokine (C-C motif) ligand 8 | 4.4 | 7.4E-04 |
| Mfsd2a | major facilitator superfamily domain containing 2A | 4.4 | 6.8E-04 |
| Nrg1 | neuregulin 1 | 4.4 | 1.8E-03 |
| Myl7 | myosin, light polypeptide 7, regulatory | 4.4 | 3.3E-04 |
| DXBay18 | DNA segment, Chr X, Baylor 18 | 4.4 | 9.5E-04 |
| Ang | angiogenin, ribonuclease, RNase A family, 5 | 4.4 | 1.2E-03 |
| Gm4684 | predicted gene 4684 | 4.2 | 7.8E-05 |
| Slc2a2 | solute carrier family 2 (facilitated glucose transporter), member 2 | 4.2 | 3.7E-04 |
| E130012A19Rik | RIKEN cDNA E130012A19 gene | 4.2 | 1.1E-04 |
| 1700010I14Rik | RIKEN cDNA 1700010I14 gene | 4.2 | 1.5E-03 |
| Gm5640 | predicted gene 5640 | 4.2 | 2.0E-03 |
| AY761185 | cDNA sequence AY761185 | 4.2 | 2.7E-03 |
| Ang4 | angiogenin, ribonuclease A family, member 4 | 4.1 | 4.5E-04 |
| Pla2g2f | phospholipase A2, group IIF | 4.1 | 2.0E-04 |
| Slc9a3 | solute carrier family 9 (sodium/hydrogen exchanger), member 3 | 4.1 | 5.2E-04 |
| Phlda1 | pleckstrin homology-like domain, family A, member 1 | 4.1 | 8.1E-04 |
| LOC790956 | 5.8S ribosomal RNA | 4.0 | 1.3E-04 |
| Derl3 | Der1-like domain family, member 3 | 4.0 | 1.1E-04 |
| D330028D13Rik | RIKEN cDNA D330028D13 gene | 4.0 | 1.1E-04 |
| Galnt14 | UDP-N-acetyl-alpha-D-galactosamine:polypeptide N-acetylgalactosaminyltransferase 14 | 3.9 | 3.6E-03 |
| Pgap1 | post-GPI attachment to proteins 1 | 3.9 | 5.6E-04 |
| St3gal6 | ST3 beta-galactoside alpha-2,3-sialyltransferase 6 | 3.8 | 3.6E-04 |

| | | | |
|---|---|---|---|
| Cd300lf | CD300 antigen like family member F | 3.8 | 5.6E-04 |
| Prl2a1 | prolactin family 2, subfamily a, member 1 | 3.8 | 1.3E-04 |
| Sh2d7 | SH2 domain containing 7 | 3.7 | 7.7E-04 |
| Trpm5 | transient receptor potential cation channel, subfamily M, member 5 | 3.7 | 1.8E-03 |
| Igh-VJ558 | immunoglobulin heavy chain (J558 family) | 3.7 | 1.3E-04 |
| Tspan6 | tetraspanin 6 | 3.7 | 1.8E-04 |
| Tg | thyroglobulin | 3.7 | 1.6E-04 |
| Tnnt3 | troponin T3, skeletal, fast | 3.7 | 1.7E-04 |
| Gm5639 | predicted pseudogene 5639 | 3.7 | 1.1E-03 |
| Mmp9 | matrix metallopeptidase 9 | 3.6 | 1.0E-03 |
| Oosp1 | oocyte secreted protein 1 | 3.6 | 1.3E-04 |
| Ly6g6d | lymphocyte antigen 6 complex, locus G6D | 3.6 | 4.1E-03 |
| B3galt5 | UDP-Gal:betaGlcNAc beta 1,3-galactosyltransferase, polypeptide 5 | 3.5 | 4.5E-03 |
| Tnfrsf12a | tumor necrosis factor receptor superfamily, member 12a | 3.5 | 1.7E-04 |
| Rnf183 | ring finger protein 183 | 3.5 | 1.1E-04 |
| Egln3 | EGL nine homolog 3 (C. elegans) | 3.5 | 3.3E-04 |
| Sval1 | seminal vesicle antigen-like 1 | 3.5 | 2.8E-04 |
| Alox5ap | arachidonate 5-lipoxygenase activating protein | 3.5 | 8.6E-04 |
| Gadd45g | growth arrest and DNA-damage-inducible 45 gamma | 3.4 | 7.9E-04 |
| Hsph1 | heat shock 105kDa/110kDa protein 1 | 3.4 | 9.2E-03 |
| Plk3 | polo-like kinase 3 (Drosophila) | 3.4 | 1.3E-03 |
| Ccr3 | chemokine (C-C motif) receptor 3 | 3.4 | 1.5E-04 |
| Il13ra2 | interleukin 13 receptor, alpha 2 | 3.4 | 5.9E-04 |
| Aif1l | allograft inflammatory factor 1-like | 3.4 | 1.3E-04 |
| Shank1 | SH3/ankyrin domain gene 1 | 3.4 | 1.3E-03 |
| Slc16a3 | solute carrier family 16 (monocarboxylic acid transporters), member 3 | 3.4 | 6.7E-04 |
| Cacna1s | calcium channel, voltage-dependent, L type, alpha 1S subunit | 3.4 | 1.7E-04 |
| Lama1 | laminin, alpha 1 | 3.4 | 7.8E-04 |
| Crlf1 | cytokine receptor-like factor 1 | 3.4 | 1.5E-03 |
| Irf4 | interferon regulatory factor 4 | 3.3 | 1.0E-03 |
| Il18r1 | interleukin 18 receptor 1 | 3.3 | 1.2E-03 |
| Tnfrsf17 | tumor necrosis factor receptor superfamily, member 17 | 3.3 | 1.3E-04 |
| Gprc5a | G protein-coupled receptor, family C, group 5, member A | 3.3 | 3.3E-04 |
| Fcrlb | Fc receptor-like B | 3.2 | 2.9E-03 |
| B3gnt7 | UDP-GlcNAc:betaGal beta-1,3-N-acetylglucosaminyltransferase 7 | 3.2 | 2.6E-04 |
| Fam40b | family with sequence similarity 40, member B | 3.2 | 1.8E-03 |
| Lrmp | lymphoid-restricted membrane protein | 3.2 | 7.9E-04 |
| Hmx2 | H6 homeobox 2 | 3.2 | 5.7E-04 |
| Celf4 | CUGBP, Elav-like family member 4 | 3.2 | 1.5E-04 |
| Obfc1 | oligonucleotide/oligosaccharide-binding fold containing 1 | 3.2 | 3.4E-04 |
| E030030I06Rik | RIKEN cDNA E030030I06 gene | 3.2 | 1.7E-04 |

| | | | |
|---|---|---|---|
| 4930405D01Rik | RIKEN cDNA 4930405D01 gene | 3.2 | 2.1E-03 |
| Gng13 | guanine nucleotide binding protein (G protein), gamma 13 | 3.2 | 1.2E-03 |
| Krtap16-7 | keratin associated protein 16-7 | 3.2 | 9.6E-04 |
| Fam178b | family with sequence similarity 178, member B | 3.2 | 1.4E-03 |
| Calca | calcitonin/calcitonin-related polypeptide, alpha | 3.2 | 2.8E-04 |
| Ccl9 | chemokine (C-C motif) ligand 9 | 3.2 | 2.6E-04 |
| Gpx2 | glutathione peroxidase 2 | 3.1 | 1.8E-04 |
| Grp | gastrin releasing peptide | 3.1 | 2.6E-03 |
| Pou2af1 | POU domain, class 2, associating factor 1 | 3.1 | 4.9E-04 |
| Mlana | melan-A | 3.1 | 1.5E-04 |
| Frmd5 | FERM domain containing 5 | 3.1 | 2.6E-04 |
| Chrm3 | cholinergic receptor, muscarinic 3, cardiac | 3.1 | 5.5E-04 |
| Pik3r5 | phosphoinositide-3-kinase, regulatory subunit 5, p101 | 3.1 | 5.5E-04 |
| Pla2g2a | phospholipase A2, group IIA (platelets, synovial fluid) | 3.1 | 2.6E-04 |
| BC051019 | cDNA sequence BC051019 | 3.1 | 1.7E-04 |
| Rgs13 | regulator of G-protein signaling 13 | 3.1 | 1.1E-03 |
| Fn1 | fibronectin 1 | 3.1 | 6.2E-04 |
| Fkbp5 | FK506 binding protein 5 | 3.1 | 5.2E-03 |
| Vpreb3 | pre-B lymphocyte gene 3 | 3.1 | 5.6E-04 |
| Gm5547 | predicted gene 5547 | 3.1 | 2.2E-04 |
| Wfikkn2 | WAP, follistatin/kazal, immunoglobulin, kunitz and netrin domain containing 2 | 3.1 | 8.5E-04 |
| Bmx | BMX non-receptor tyrosine kinase | 3.1 | 1.0E-03 |
| Avil | advillin | 3.0 | 1.1E-03 |
| C2cd4b | C2 calcium-dependent domain containing 4B | 3.0 | 2.2E-03 |
| Siglec5 | sialic acid binding Ig-like lectin 5 | 3.0 | 4.0E-03 |
| Tcp10b | t-complex protein 10b | 3.0 | 4.7E-03 |
| 5430405G05Rik | RIKEN cDNA 5430405G05 gene | 3.0 | 2.8E-03 |
| Gch1 | GTP cyclohydrolase 1 | 3.0 | 1.8E-03 |
| Lbh | limb-bud and heart | 3.0 | 1.6E-04 |
| C3 | complement component 3 | 3.0 | 3.3E-03 |
| Rgs22 | regulator of G-protein signalling 22 | 3.0 | 5.4E-04 |
| Tnfrsf21 | tumor necrosis factor receptor superfamily, member 21 | 3.0 | 1.8E-04 |
| Col6a5 | collagen, type VI, alpha 5 | 3.0 | 3.7E-04 |
| Eaf2 | ELL associated factor 2 | 3.0 | 2.8E-04 |
| 9030619P08Rik | RIKEN cDNA 9030619P08 gene | 3.0 | 4.0E-04 |
| C4bp | complement component 4 binding protein | 3.0 | 1.1E-03 |
| S100a14 | S100 calcium binding protein A14 | 3.0 | 1.8E-04 |
| Otud3 | OTU domain containing 3 | 3.0 | 5.7E-04 |
| Epgn | epithelial mitogen | 3.0 | 3.5E-04 |
| Hck | hemopoietic cell kinase | 3.0 | 1.1E-03 |
| Ankrd22 | ankyrin repeat domain 22 | 3.0 | 5.3E-04 |
| Ccdc129 | coiled-coil domain containing 129 | 2.9 | 4.3E-04 |
| Matk | megakaryocyte-associated tyrosine kinase | 2.9 | 3.4E-03 |
| Olfr1252 | olfactory receptor 1252 | 2.9 | 9.0E-04 |
| Tnfrsf13b | tumor necrosis factor receptor superfamily, member 13b | 2.9 | 2.0E-04 |
| Hmx3 | H6 homeobox 3 | 2.9 | 7.7E-04 |

| | | | |
|---|---|---|---|
| Mboat1 | membrane bound O-acyltransferase domain containing 1 | 2.9 | 1.5E-03 |
| Serpina3n | serine (or cysteine) peptidase inhibitor, clade A, member 3N | 2.9 | 9.7E-04 |
| Fer1l4 | fer-1-like 4 (C. elegans) | 2.9 | 1.4E-03 |
| Slc20a1 | solute carrier family 20, member 1 | 2.9 | 3.8E-04 |
| Gm6934 | predicted gene 6934 | 2.9 | 3.3E-04 |
| Ces1c | carboxylesterase 1C | 2.9 | 2.4E-03 |
| Fam46c | family with sequence similarity 46, member C | 2.9 | 8.5E-04 |
| Hk2 | hexokinase 2 | 2.9 | 5.4E-04 |
| 4930444M15Rik | RIKEN cDNA 4930444M15 gene | 2.9 | 4.2E-04 |
| Vmn2r26 | vomeronasal 2, receptor 26 | 2.8 | 2.5E-03 |
| Ccl7 | chemokine (C-C motif) ligand 7 | 2.8 | 1.1E-03 |
| Slc28a1 | solute carrier family 28 (sodium-coupled nucleoside transporter), member 1 | 2.8 | 5.5E-03 |
| Plxna2 | plexin A2 | 2.8 | 2.6E-04 |
| Nfasc | neurofascin | 2.8 | 3.0E-04 |
| 2010002N04Rik | RIKEN cDNA 2010002N04 gene | 2.8 | 3.3E-04 |
| Gramd2 | GRAM domain containing 2 | 2.8 | 1.2E-03 |
| Gfi1b | growth factor independent 1B | 2.8 | 3.9E-03 |
| Bhmt2 | betaine-homocysteine methyltransferase 2 | 2.8 | 3.4E-03 |
| Hsd11b1 | hydroxysteroid 11-beta dehydrogenase 1 | 2.8 | 5.4E-04 |
| Slc2a1 | solute carrier family 2 (facilitated glucose transporter), member 1 | 2.8 | 3.4E-04 |
| Serpina3f | serine (or cysteine) peptidase inhibitor, clade A, member 3F | 2.7 | 6.1E-03 |
| Dclk1 | doublecortin-like kinase 1 | 2.7 | 2.2E-03 |
| Pygl | liver glycogen phosphorylase | 2.7 | 4.5E-04 |
| 1810046K07Rik | RIKEN cDNA 1810046K07 gene | 2.7 | 6.9E-04 |
| Mmp7 | matrix metallopeptidase 7 | 2.7 | 1.5E-03 |
| Gm2696 | predicted gene 2696 | 2.7 | 3.2E-04 |
| Igj | immunoglobulin joining chain | 2.7 | 5.5E-04 |
| Pde2a | phosphodiesterase 2A, cGMP-stimulated | 2.7 | 6.9E-04 |
| Cartpt | CART prepropeptide | 2.7 | 6.3E-03 |
| Cd24a | CD24a antigen | 2.7 | 4.5E-04 |
| A630081J09Rik | RIKEN cDNA A630081J09 gene | 2.7 | 4.5E-04 |
| Spon1 | spondin 1, (f-spondin) extracellular matrix protein | 2.7 | 6.8E-04 |
| Adamts4 | a disintegrin-like and metallopeptidase (reprolysin type) with thrombospondin type 1 motif, 4 | 2.7 | 5.0E-03 |
| Car8 | carbonic anhydrase 8 | 2.7 | 5.2E-03 |
| Sh2d6 | SH2 domain containing 6 | 2.7 | 2.0E-03 |
| Igf1 | insulin-like growth factor 1 | 2.7 | 1.2E-03 |
| Phlda2 | pleckstrin homology-like domain, family A, member 2 | 2.7 | 7.5E-04 |
| Mfap4 | microfibrillar-associated protein 4 | 2.7 | 4.0E-03 |
| Alox15 | arachidonate 15-lipoxygenase | 2.7 | 1.0E-03 |
| Gpr55 | G protein-coupled receptor 55 | 2.7 | 3.4E-04 |
| Tuba8 | tubulin, alpha 8 | 2.7 | 1.2E-03 |
| Spdef | SAM pointed domain containing ets transcription factor | 2.6 | 4.0E-04 |

| | | | |
|---|---|---|---|
| Ear10 | eosinophil-associated, ribonuclease A family, member 10 | 2.6 | 4.9E-04 |
| Il18 | interleukin 18 | 2.6 | 3.2E-03 |
| Diras2 | DIRAS family, GTP-binding RAS-like 2 | 2.6 | 9.8E-04 |
| Lin52 | lin-52 homolog (C. elegans) | 2.6 | 1.2E-03 |
| Svep1 | sushi, von Willebrand factor type A, EGF and pentraxin domain containing 1 | 2.6 | 7.7E-04 |
| Wtip | WT1-interacting protein | 2.6 | 5.2E-04 |
| Meis3 | Meis homeobox 3 | 2.6 | 1.1E-03 |
| Anxa10 | annexin A10 | 2.6 | 7.7E-03 |
| Serpina3g | serine (or cysteine) peptidase inhibitor, clade A, member 3G | 2.6 | 1.2E-03 |
| Il33 | interleukin 33 | 2.6 | 2.8E-03 |
| Klk1b8 | kallikrein 1-related peptidase b8 | 2.6 | 3.3E-04 |
| Prom2 | prominin 2 | 2.6 | 4.9E-03 |
| Serpinb5 | serine (or cysteine) peptidase inhibitor, clade B, member 5 | 2.6 | 7.4E-04 |
| Ptgs2 | prostaglandin-endoperoxide synthase 2 | 2.6 | 8.8E-04 |
| Fetub | fetuin beta | 2.6 | 3.0E-04 |
| Il17rb | interleukin 17 receptor B | 2.6 | 4.2E-04 |
| Slc17a9 | solute carrier family 17, member 9 | 2.6 | 3.8E-04 |
| Itgal | integrin alpha L | 2.6 | 6.9E-04 |
| Ccl11 | chemokine (C-C motif) ligand 11 | 2.5 | 8.5E-03 |
| 4921516I12Rik | RIKEN cDNA 4921516I12 gene | 2.5 | 6.7E-04 |
| Olfr374 | olfactory receptor 374 | 2.5 | 4.0E-03 |
| Ly6g6f | lymphocyte antigen 6 complex, locus G6F | 2.5 | 4.5E-03 |
| Hoxa3 | homeobox A3 | 2.5 | 8.4E-04 |
| Habp2 | hyaluronic acid binding protein 2 | 2.5 | 5.9E-03 |
| Klk1b22 | kallikrein 1-related peptidase b22 | 2.5 | 5.7E-04 |
| Rbp2 | retinol binding protein 2, cellular | 2.5 | 3.3E-04 |
| Klk1b24 | kallikrein 1-related peptidase b24 | 2.5 | 7.2E-04 |
| Tesc | tescalcin | 2.5 | 1.1E-03 |
| Col7a1 | collagen, type VII, alpha 1 | 2.5 | 2.2E-03 |
| Sycn | syncollin | 2.5 | 3.7E-04 |
| Klk1b4 | kallikrein 1-related pepidase b4 | 2.5 | 5.7E-04 |
| Tmem181b-ps | transmembrane protein 181B, pseudogene | 2.5 | 7.1E-04 |
| Hs3st1 | heparan sulfate (glucosamine) 3-O-sulfotransferase 1 | 2.5 | 1.2E-03 |
| Creld2 | cysteine-rich with EGF-like domains 2 | 2.4 | 4.5E-03 |
| Vav1 | vav 1 oncogene | 2.4 | 1.2E-03 |
| Hif1a | hypoxia inducible factor 1, alpha subunit | 2.4 | 2.0E-03 |
| Nr4a2 | nuclear receptor subfamily 4, group A, member 2 | 2.4 | 6.4E-04 |
| Csrnp1 | cysteine-serine-rich nuclear protein 1 | 2.4 | 5.4E-04 |
| Glipr1 | GLI pathogenesis-related 1 (glioma) | 2.4 | 4.0E-04 |
| Cela1 | chymotrypsin-like elastase family, member 1 | 2.4 | 6.3E-04 |
| Sucnr1 | succinate receptor 1 | 2.4 | 3.5E-03 |
| Fam73a | family with sequence similarity 73, member A | 2.4 | 5.1E-04 |
| A4gnt | alpha-1,4-N-acetylglucosaminyltransferase | 2.4 | 6.1E-04 |
| Fut1 | fucosyltransferase 1 | 2.4 | 2.0E-03 |
| Il11 | interleukin 11 | 2.4 | 3.7E-03 |

| | | | |
|---|---|---|---|
| Tle1 | transducin-like enhancer of split 1, homolog of Drosophila E(spl) | 2.4 | 5.2E-03 |
| Ear2 | eosinophil-associated, ribonuclease A family, member 2 | 2.4 | 2.0E-03 |
| Tmprss2 | transmembrane protease, serine 2 | 2.4 | 6.4E-04 |
| Socs3 | suppressor of cytokine signaling 3 | 2.4 | 2.9E-03 |
| Jakmip3 | janus kinase and microtubule interacting protein 3 | 2.4 | 7.9E-04 |
| Plaur | plasminogen activator, urokinase receptor | 2.4 | 5.3E-04 |
| Lypd2 | Ly6/Plaur domain containing 2 | 2.4 | 1.3E-03 |
| Casp3 | caspase 3 | 2.4 | 1.0E-03 |
| Spcs3 | signal peptidase complex subunit 3 homolog (S. cerevisiae) | 2.4 | 6.9E-04 |
| Gm11428 | predicted gene 11428 | 2.4 | 9.6E-04 |
| Tnfrsf13c | tumor necrosis factor receptor superfamily, member 13c | 2.4 | 1.5E-03 |
| Dusp4 | dual specificity phosphatase 4 | 2.4 | 5.4E-03 |
| Lin7a | lin-7 homolog A (C. elegans) | 2.4 | 3.9E-03 |
| Krt23 | keratin 23 | 2.3 | 1.6E-03 |
| Myadm | myeloid-associated differentiation marker | 2.3 | 6.9E-04 |
| Pou2f2 | POU domain, class 2, transcription factor 2 | 2.3 | 4.7E-04 |
| C130026I21Rik | RIKEN cDNA C130026I21 gene | 2.3 | 5.7E-04 |
| Serp2 | stress-associated endoplasmic reticulum protein family member 2 | 2.3 | 2.3E-03 |
| Spib | Spi-B transcription factor (Spi-1/PU.1 related) | 2.3 | 5.2E-03 |
| a | nonagouti | 2.3 | 1.4E-03 |
| Slc41a1 | solute carrier family 41, member 1 | 2.3 | 5.8E-04 |
| Klk1b3 | kallikrein 1-related peptidase b3 | 2.3 | 5.5E-04 |
| Cd79a | CD79A antigen (immunoglobulin-associated alpha) | 2.3 | 3.0E-03 |
| Fam101a | family with sequence similarity 101, member A | 2.3 | 3.5E-03 |
| Gata3 | GATA binding protein 3 | 2.3 | 6.9E-04 |
| Grpr | gastrin releasing peptide receptor | 2.3 | 2.6E-03 |
| Nupr1 | nuclear protein 1 | 2.3 | 4.2E-03 |
| Cmah | cytidine monophospho-N-acetylneuraminic acid hydroxylase | 2.3 | 6.7E-04 |
| Proc | protein C | 2.3 | 1.9E-03 |
| Timp1 | tissue inhibitor of metalloproteinase 1 | 2.3 | 5.2E-03 |
| Il4 | interleukin 4 | 2.3 | 5.3E-04 |
| Ldhc | lactate dehydrogenase C | 2.3 | 1.6E-03 |
| Hpd | 4-hydroxyphenylpyruvic acid dioxygenase | 2.3 | 8.8E-04 |
| Gm10325 | predicted gene 10325 | 2.3 | 6.2E-03 |
| Galnt5 | UDP-N-acetyl-alpha-D-galactosamine:polypeptide N-acetylgalactosaminyltransferase 5 | 2.3 | 2.2E-03 |
| Gsr | glutathione reductase | 2.3 | 1.4E-03 |
| Pla2g7 | phospholipase A2, group VII (platelet-activating factor acetylhydrolase, plasma) | 2.3 | 1.5E-03 |
| Klk1 | kallikrein 1 | 2.3 | 6.1E-04 |
| Socs1 | suppressor of cytokine signaling 1 | 2.3 | 2.2E-03 |
| Bhlha15 | basic helix-loop-helix family, member a15 | 2.3 | 9.5E-04 |

| | | | |
|---|---|---|---|
| Pik3cg | phosphoinositide-3-kinase, catalytic, gamma polypeptide | 2.2 | 1.2E-03 |
| LOC100504608 | protein FAM119B-like | 2.2 | 5.3E-04 |
| C6 | complement component 6 | 2.2 | 9.3E-04 |
| 4930583H14Rik | RIKEN cDNA 4930583H14 gene | 2.2 | 5.5E-03 |
| Neb | nebulin | 2.2 | 6.9E-04 |
| Il5ra | interleukin 5 receptor, alpha | 2.2 | 5.9E-04 |
| Rasgrf2 | RAS protein-specific guanine nucleotide-releasing factor 2 | 2.2 | 1.2E-03 |
| Acot7 | acyl-CoA thioesterase 7 | 2.2 | 1.2E-03 |
| Sidt1 | SID1 transmembrane family, member 1 | 2.2 | 5.8E-04 |
| Odf2l | outer dense fiber of sperm tails 2-like | 2.2 | 1.1E-03 |
| Fam167b | family with sequence similarity 167, member B | 2.2 | 7.4E-03 |
| Ckb | creatine kinase, brain | 2.2 | 1.6E-03 |
| Slc12a6 | solute carrier family 12, member 6 | 2.2 | 1.4E-03 |
| Cd79b | CD79B antigen | 2.2 | 5.4E-03 |
| Mecom | MDS1 and EVI1 complex locus | 2.2 | 1.7E-03 |
| Nucb2 | nucleobindin 2 | 2.2 | 1.2E-03 |
| Fcrla | Fc receptor-like A | 2.2 | 7.4E-04 |
| Tmed6 | transmembrane emp24 protein transport domain containing 6 | 2.2 | 4.3E-03 |
| Mvd | mevalonate (diphospho) decarboxylase | 2.2 | 1.1E-03 |
| Epha8 | Eph receptor A8 | 2.2 | 2.6E-03 |
| H19 | H19 fetal liver mRNA | 2.2 | 7.9E-04 |
| Prss50 | protease, serine, 50 | 2.2 | 9.1E-03 |
| Sh2b2 | SH2B adaptor protein 2 | 2.2 | 6.0E-03 |
| Dusp6 | dual specificity phosphatase 6 | 2.2 | 7.4E-04 |
| Cd44 | CD44 antigen | 2.2 | 6.7E-04 |
| Samd5 | sterile alpha motif domain containing 5 | 2.2 | 2.3E-03 |
| B430306N03Rik | RIKEN cDNA B430306N03 gene | 2.2 | 2.8E-03 |
| Olfr316 | olfactory receptor 316 | 2.2 | 3.9E-03 |
| 1810010D01Rik | RIKEN cDNA 1810010D01 gene | 2.2 | 2.5E-03 |
| Krt17 | keratin 17 | 2.2 | 3.4E-03 |
| Col6a2 | collagen, type VI, alpha 2 | 2.2 | 6.7E-03 |
| 6430548M08Rik | RIKEN cDNA 6430548M08 gene | 2.2 | 7.0E-04 |
| Dok2 | docking protein 2 | 2.2 | 4.2E-03 |
| Gkn3 | gastrokine 3 | 2.2 | 7.2E-04 |
| Gprc5c | G protein-coupled receptor, family C, group 5, member C | 2.2 | 5.1E-03 |
| Tmem121 | transmembrane protein 121 | 2.2 | 2.6E-03 |
| Prss35 | protease, serine, 35 | 2.2 | 9.0E-04 |
| Ptpro | protein tyrosine phosphatase, receptor type, O | 2.2 | 2.3E-03 |
| BB086117 | expressed sequence BB086117 | 2.2 | 1.2E-03 |
| Cyp4f18 | cytochrome P450, family 4, subfamily f, polypeptide 18 | 2.1 | 1.4E-03 |
| B020031M17Rik | RIKEN cDNA B020031M17 gene | 2.1 | 9.1E-03 |
| Tnk2 | tyrosine kinase, non-receptor, 2 | 2.1 | 6.6E-04 |
| Rac2 | RAS-related C3 botulinum substrate 2 | 2.1 | 1.2E-03 |
| Dio2 | deiodinase, iodothyronine, type II | 2.1 | 7.4E-04 |
| Klk1b21 | kallikrein 1-related peptidase b21 | 2.1 | 1.6E-03 |

| | | | |
|---|---|---|---|
| Pdcd1lg2 | programmed cell death 1 ligand 2 | 2.1 | 6.6E-04 |
| Il6 | interleukin 6 | 2.1 | 8.3E-04 |
| Sp140 | Sp140 nuclear body protein | 2.1 | 1.4E-03 |
| Lilra6 | leukocyte immunoglobulin-like receptor, subfamily A (with TM domain), member 6 | 2.1 | 6.7E-04 |
| Mmp10 | matrix metallopeptidase 10 | 2.1 | 1.1E-03 |
| Gata1 | GATA binding protein 1 | 2.1 | 7.0E-04 |
| Tifa | TRAF-interacting protein with forkhead-associated domain | 2.1 | 1.4E-03 |
| 2010109I03Rik | RIKEN cDNA 2010109I03 gene | 2.1 | 5.7E-03 |
| Fkbp1a | FK506 binding protein 1a | 2.1 | 9.4E-03 |
| Stac2 | SH3 and cysteine rich domain 2 | 2.1 | 1.5E-03 |
| Rbp3 | retinol binding protein 3, interstitial | 2.1 | 1.4E-03 |
| Ccr10 | chemokine (C-C motif) receptor 10 | 2.1 | 3.3E-03 |
| Hgfac | hepatocyte growth factor activator | 2.1 | 7.2E-04 |
| Samsn1 | SAM domain, SH3 domain and nuclear localization signals, 1 | 2.1 | 7.4E-04 |
| Azgp1 | alpha-2-glycoprotein 1, zinc | 2.1 | 2.6E-03 |
| 9130008F23Rik | RIKEN cDNA 9130008F23 gene | 2.1 | 2.1E-03 |
| Kremen2 | kringle containing transmembrane protein 2 | 2.1 | 7.5E-04 |
| Ergic1 | endoplasmic reticulum-golgi intermediate compartment (ERGIC) 1 | 2.1 | 8.3E-04 |
| Ubxn11 | UBX domain protein 11 | 2.1 | 1.5E-03 |
| Ethe1 | ethylmalonic encephalopathy 1 | 2.1 | 1.3E-03 |
| Ociad2 | OCIA domain containing 2 | 2.1 | 1.1E-03 |
| Cpa3 | carboxypeptidase A3, mast cell | 2.1 | 9.7E-04 |
| Kctd12 | potassium channel tetramerisation domain containing 12 | 2.1 | 9.3E-04 |
| Rbp1 | retinol binding protein 1, cellular | 2.1 | 1.8E-03 |
| Lypd3 | Ly6/Plaur domain containing 3 | 2.1 | 4.0E-03 |
| Klk1b26 | kallikrein 1-related petidase b26 | 2.1 | 9.8E-04 |
| Arl5b | ADP-ribosylation factor-like 5B | 2.1 | 1.2E-03 |
| Egfbp2 | epidermal growth factor binding protein type B | 2.1 | 1.8E-03 |
| C4b | complement component 4B (Childo blood group) | 2.1 | 1.6E-03 |
| Nkx3-2 | NK3 homeobox 2 | 2.1 | 2.5E-03 |
| St3gal3 | ST3 beta-galactoside alpha-2,3-sialyltransferase 3 | 2.1 | 1.2E-03 |
| B4galt1 | UDP-Gal:betaGlcNAc beta 1,4- galactosyltransferase, polypeptide 1 | 2.1 | 9.4E-04 |
| Pla2g10 | phospholipase A2, group X | 2.1 | 2.3E-03 |
| Vill | villin-like | 2.1 | 1.9E-03 |
| Ear1 | eosinophil-associated, ribonuclease A family, member 1 | 2.1 | 1.4E-03 |
| S100a1 | S100 calcium binding protein A1 | 2.1 | 8.4E-04 |
| Sulf1 | sulfatase 1 | 2.1 | 8.6E-03 |
| Apoc2 | apolipoprotein C-II | 2.1 | 2.8E-03 |
| Il25 | interleukin 25 | 2.1 | 6.8E-03 |
| Fzd9 | frizzled homolog 9 (Drosophila) | 2.1 | 2.6E-03 |
| Tnfsf13b | tumor necrosis factor (ligand) superfamily, member 13b | 2.1 | 1.3E-03 |

| | | | |
|---|---|---|---|
| Ndufaf4 | NADH dehydrogenase (ubiquinone) 1 alpha subcomplex, assembly factor 4 | 2.1 | 5.2E-03 |
| Etv4 | ets variant gene 4 (E1A enhancer binding protein, E1AF) | 2.1 | 2.0E-03 |
| Dgki | diacylglycerol kinase, iota | 2.1 | 8.5E-03 |
| Nrgn | neurogranin | 2.1 | 3.6E-03 |
| Fxyd3 | FXYD domain-containing ion transport regulator 3 | 2.0 | 1.5E-03 |
| Krt16 | keratin 16 | 2.0 | 1.6E-03 |
| Cd2 | CD2 antigen | 2.0 | 1.1E-03 |
| Mei1 | meiosis defective 1 | 2.0 | 3.2E-03 |
| Icos | inducible T-cell co-stimulator | 2.0 | 1.6E-03 |
| Muc4 | mucin 4, ASGP | 2.0 | 8.4E-03 |
| Tmem29 | transmembrane protein 29 | 2.0 | 3.4E-03 |
| Slit1 | slit homolog 1 (Drosophila) | 2.0 | 6.4E-03 |
| F5 | coagulation factor V | 2.0 | 9.6E-04 |
| Gusb | glucuronidase, beta | 2.0 | 1.1E-03 |
| Snrnp25 | small nuclear ribonucleoprotein 25 (U11/U12) | 2.0 | 1.8E-03 |
| Rep15 | RAB15 effector protein | 2.0 | 1.2E-03 |
| Mfsd4 | major facilitator superfamily domain containing 4 | 2.0 | 6.0E-03 |
| Smox | spermine oxidase | 2.0 | 1.6E-03 |
| Pla2g12a | phospholipase A2, group XIIA | 2.0 | 1.0E-03 |
| Adcy4 | adenylate cyclase 4 | 2.0 | 8.8E-03 |
| 1700007K09Rik | RIKEN cDNA 1700007K09 gene | 2.0 | 2.4E-03 |
| Il5 | interleukin 5 | 2.0 | 1.2E-03 |
| Slk | STE20-like kinase (yeast) | 2.0 | 4.5E-03 |
| Sox9 | SRY-box containing gene 9 | 2.0 | 1.1E-03 |
| Gapt | Grb2-binding adaptor, transmembrane | 2.0 | 4.6E-03 |

Table S2. Mouse genes downregulated in ileum of *Plcb3*$^{-/-}$ mice

| Gene symbol | Gene name | Fold change (KO vs WT) | q-value |
|---|---|---|---|
| Plcb3 | phospholipase C, beta 3 | -278.2 | 5.1E-07 |
| Syt10 | synaptotagmin X | -32.6 | 2.8E-05 |
| Lct | lactase | -24.8 | 1.3E-04 |
| Slc5a12 | solute carrier family 5 (sodium/glucose cotransporter), member 12 | -17.6 | 8.0E-05 |
| G6pc | glucose-6-phosphatase, catalytic | -17.3 | 9.3E-04 |
| Dnase1 | deoxyribonuclease I | -16.5 | 1.8E-03 |
| Plb1 | phospholipase B1 | -15.8 | 2.4E-04 |
| Rdh7 | retinol dehydrogenase 7 | -11.1 | 1.6E-04 |
| Cyp2b10 | cytochrome P450, family 2, subfamily b, polypeptide 10 | -10.8 | 5.2E-04 |
| Cyp2d40 | cytochrome P450, family 2, subfamily d, polypeptide 40 | -10.2 | 7.8E-05 |
| Olfr165 | olfactory receptor 165 | -10.2 | 3.1E-05 |
| Ccl5 | chemokine (C-C motif) ligand 5 | -9.5 | 2.3E-04 |
| Aqp3 | aquaporin 3 | -9.4 | 2.5E-04 |
| Reg3a | regenerating islet-derived 3 alpha | -9.4 | 9.3E-05 |
| Enpp7 | ectonucleotide pyrophosphatase/phosphodiesterase 7 | -9.4 | 7.8E-05 |
| Slc34a2 | solute carrier family 34 (sodium phosphate), member 2 | -9.2 | 1.9E-04 |
| BC089597 | cDNA sequence BC089597 | -8.4 | 5.1E-04 |
| Aadac | arylacetamide deacetylase (esterase) | -8.2 | 4.6E-05 |
| Ces1g | carboxylesterase 1G | -7.2 | 1.3E-04 |
| Leap2 | liver-expressed antimicrobial peptide 2 | -7.2 | 1.0E-03 |
| Slc5a4b | solute carrier family 5 (neutral amino acid transporters, system A), member 4b | -7.2 | 7.2E-05 |
| Ocm | oncomodulin | -7.1 | 4.6E-05 |
| Trpm6 | transient receptor potential cation channel, subfamily M, member 6 | -6.6 | 3.2E-03 |
| Serpina1c | serine (or cysteine) peptidase inhibitor, clade A, member 1C | -6.2 | 1.3E-04 |
| Serpina1a | serine (or cysteine) peptidase inhibitor, clade A, member 1A | -6.2 | 1.6E-04 |
| Gzmb | granzyme B | -6.2 | 4.6E-05 |
| Serpina1e | serine (or cysteine) peptidase inhibitor, clade A, member 1E | -6.1 | 1.4E-04 |
| 2010005H15Rik | RIKEN cDNA 2010005H15 gene | -6.1 | 7.2E-05 |
| Mme | membrane metallo endopeptidase | -6.1 | 6.4E-05 |
| Acot12 | acyl-CoA thioesterase 12 | -6.0 | 5.8E-05 |
| Serpina1d | serine (or cysteine) peptidase inhibitor, clade A, member 1D | -6.0 | 1.3E-04 |
| Hsd17b13 | hydroxysteroid (17-beta) dehydrogenase 13 | -6.0 | 5.0E-05 |
| E130116L18Rik | RIKEN cDNA E130116L18 gene | -5.9 | 1.3E-04 |
| Igf2as | insulin-like growth factor 2, antisense | -5.8 | 5.7E-05 |
| Angptl4 | angiopoietin-like 4 | -5.6 | 3.8E-04 |
| Ugt2b5 | UDP glucuronosyltransferase 2 family, polypeptide B5 | -5.5 | 6.7E-05 |
| Gp2 | glycoprotein 2 (zymogen granule membrane) | -5.5 | 6.4E-04 |

| | | | |
|---|---|---|---|
| Mt2 | metallothionein 2 | -5.5 | 9.3E-05 |
| Ada | adenosine deaminase | -5.3 | 9.9E-05 |
| Slc6a20a | solute carrier family 6 (neurotransmitter transporter), member 20A | -5.3 | 2.6E-04 |
| Cyp4v3 | cytochrome P450, family 4, subfamily v, polypeptide 3 | -5.3 | 1.1E-04 |
| Ugt2b38 | UDP glucuronosyltransferase 2 family, polypeptide B38 | -5.2 | 8.0E-05 |
| Akr1b7 | aldo-keto reductase family 1, member B7 | -5.1 | 2.6E-04 |
| 1700057G04Rik | RIKEN cDNA 1700057G04 gene | -5.0 | 1.2E-04 |
| Spsb4 | splA/ryanodine receptor domain and SOCS box containing 4 | -4.9 | 4.4E-04 |
| Gzma | granzyme A | -4.9 | 3.4E-04 |
| Otop2 | otopetrin 2 | -4.9 | 1.3E-04 |
| Cyp3a57 | cytochrome P450, family 3, subfamily a, polypeptide 57 | -4.9 | 1.2E-04 |
| 1810065E05Rik | RIKEN cDNA 1810065E05 gene | -4.9 | 5.6E-03 |
| Ttc36 | tetratricopeptide repeat domain 36 | -4.9 | 5.1E-04 |
| Cyp2d12 | cytochrome P450, family 2, subfamily d, polypeptide 12 | -4.8 | 7.0E-05 |
| Ugt2b36 | UDP glucuronosyltransferase 2 family, polypeptide B36 | -4.8 | 6.8E-05 |
| Ubd | ubiquitin D | -4.8 | 7.8E-05 |
| Ces1f | carboxylesterase 1F | -4.7 | 2.8E-04 |
| Gpr133 | G protein-coupled receptor 133 | -4.6 | 1.1E-04 |
| Cyp2d26 | cytochrome P450, family 2, subfamily d, polypeptide 26 | -4.6 | 2.3E-04 |
| Cmpk2 | cytidine monophosphate (UMP-CMP) kinase 2, mitochondrial | -4.4 | 5.9E-04 |
| Cldn8 | claudin 8 | -4.4 | 2.9E-04 |
| Hsd3b3 | hydroxy-delta-5-steroid dehydrogenase, 3 beta- and steroid delta-isomerase 3 | -4.4 | 7.2E-05 |
| Ifit1 | interferon-induced protein with tetratricopeptide repeats 1 | -4.4 | 1.7E-04 |
| Rnf208 | ring finger protein 208 | -4.4 | 1.0E-03 |
| Ugt2b37 | UDP glucuronosyltransferase 2 family, polypeptide B37 | -4.3 | 1.8E-04 |
| Fras1 | Fraser syndrome 1 homolog (human) | -4.3 | 2.8E-04 |
| Gm5485 | predicted gene 5485 | -4.3 | 3.2E-04 |
| Cyp3a25 | cytochrome P450, family 3, subfamily a, polypeptide 25 | -4.2 | 1.5E-04 |
| Cyp3a59 | cytochrome P450, subfamily 3A, polypeptide 59 | -4.2 | 1.2E-04 |
| Cubn | cubilin (intrinsic factor-cobalamin receptor) | -4.1 | 3.3E-04 |
| Plk5 | polo-like kinase 5 (Drosophila) | -4.1 | 1.8E-04 |
| 0610008F07Rik | RIKEN cDNA 0610008F07 gene | -4.1 | 1.9E-03 |
| Cyp3a11 | cytochrome P450, family 3, subfamily a, polypeptide 11 | -4.1 | 2.4E-04 |
| BC100530 | cDNA sequence BC100530 | -4.0 | 1.3E-04 |
| Gal3st2 | galactose-3-O-sulfotransferase 2 | -4.0 | 1.6E-04 |
| Apoc3 | apolipoprotein C-III | -3.9 | 8.5E-04 |

| | | | |
|---|---|---|---|
| Hsd3b2 | hydroxy-delta-5-steroid dehydrogenase, 3 beta- and steroid delta-isomerase 2 | -3.9 | 1.1E-04 |
| Cpn1 | carboxypeptidase N, polypeptide 1 | -3.9 | 2.9E-04 |
| Gm5549 | predicted gene 5549 | -3.9 | 4.6E-03 |
| Mal | myelin and lymphocyte protein, T-cell differentiation protein | -3.8 | 3.8E-04 |
| Pdzk1 | PDZ domain containing 1 | -3.8 | 1.3E-03 |
| Cat | catalase | -3.8 | 3.3E-04 |
| Aldh1a7 | aldehyde dehydrogenase family 1, subfamily A7 | -3.8 | 1.0E-04 |
| Slc5a6 | solute carrier family 5 (sodium-dependent vitamin transporter), member 6 | -3.8 | 2.9E-03 |
| Gbp6 | guanylate binding protein 6 | -3.7 | 7.2E-04 |
| Cyp2d22 | cytochrome P450, family 2, subfamily d, polypeptide 22 | -3.7 | 2.9E-04 |
| Pip5k1a | phosphatidylinositol-4-phosphate 5-kinase, type 1 alpha | -3.6 | 2.8E-04 |
| Slc34a3 | solute carrier family 34 (sodium phosphate), member 3 | -3.6 | 7.4E-04 |
| AW011956 | expressed sequence AW011956 | -3.6 | 3.4E-03 |
| 1700011H14Rik | RIKEN cDNA 1700011H14 gene | -3.6 | 2.8E-04 |
| Susd2 | sushi domain containing 2 | -3.5 | 4.3E-03 |
| Slc28a2 | solute carrier family 28 (sodium-coupled nucleoside transporter), member 2 | -3.5 | 1.8E-04 |
| Adh4 | alcohol dehydrogenase 4 (class II), pi polypeptide | -3.5 | 4.1E-04 |
| Dfna5 | deafness, autosomal dominant 5 (human) | -3.5 | 3.5E-04 |
| Cd7 | CD7 antigen | -3.5 | 3.4E-04 |
| Inpp5e | inositol polyphosphate-5-phosphatase E | -3.5 | 1.8E-04 |
| Gm9926 | predicted gene 9926 | -3.4 | 7.4E-04 |
| Abca8b | ATP-binding cassette, sub-family A (ABC1), member 8b | -3.4 | 7.9E-04 |
| Sgk1 | serum/glucocorticoid regulated kinase 1 | -3.4 | 6.0E-03 |
| Cyp3a16 | cytochrome P450, family 3, subfamily a, polypeptide 16 | -3.4 | 7.9E-04 |
| Kcnu1 | potassium channel, subfamily U, member 1 | -3.4 | 6.4E-04 |
| Cyp4f16 | cytochrome P450, family 4, subfamily f, polypeptide 16 | -3.4 | 1.3E-04 |
| Aldh1a1 | aldehyde dehydrogenase family 1, subfamily A1 | -3.4 | 2.6E-04 |
| Apol9b | apolipoprotein L 9b | -3.4 | 5.1E-04 |
| Mfsd7b | major facilitator superfamily domain containing 7B | -3.3 | 3.5E-04 |
| Apol9a | apolipoprotein L 9a | -3.3 | 4.1E-04 |
| Zdhhc19 | zinc finger, DHHC domain containing 19 | -3.3 | 1.7E-03 |
| Bst1 | bone marrow stromal cell antigen 1 | -3.2 | 4.9E-03 |
| Spata2l | spermatogenesis associated 2-like | -3.2 | 3.0E-04 |
| Rhebl1 | Ras homolog enriched in brain like 1 | -3.2 | 3.0E-04 |
| Fbp1 | fructose bisphosphatase 1 | -3.2 | 1.7E-04 |
| Sectm1b | secreted and transmembrane 1B | -3.2 | 5.6E-04 |
| Cyp2d11 | cytochrome P450, family 2, subfamily d, polypeptide 11 | -3.2 | 3.3E-04 |
| Agmo | alkylglycerol monooxygenase | -3.2 | 1.7E-04 |
| Hao2 | hydroxyacid oxidase 2 | -3.2 | 2.8E-03 |

| | | | |
|---|---|---|---|
| Rtp4 | receptor transporter protein 4 | -3.2 | 4.2E-04 |
| Tchh | trichohyalin | -3.2 | 1.4E-04 |
| Slc25a45 | solute carrier family 25, member 45 | -3.1 | 3.3E-03 |
| Gm10046 | predicted gene 10046 | -3.1 | 3.1E-04 |
| Cdkl1 | cyclin-dependent kinase-like 1 (CDC2-related kinase) | -3.1 | 1.5E-03 |
| Trdmt1 | tRNA aspartic acid methyltransferase 1 | -3.1 | 3.6E-04 |
| Slc47a1 | solute carrier family 47, member 1 | -3.1 | 6.4E-04 |
| Gm14446 | predicted gene 14446 | -3.1 | 5.7E-04 |
| Rsad2 | radical S-adenosyl methionine domain containing 2 | -3.1 | 1.7E-04 |
| Amn | amnionless | -3.1 | 6.7E-04 |
| Cyp2c54 | cytochrome P450, family 2, subfamily c, polypeptide 54 | -3.1 | 1.9E-04 |
| Slc38a8 | solute carrier family 38, member 8 | -3.0 | 3.0E-04 |
| Ephx2 | epoxide hydrolase 2, cytoplasmic | -3.0 | 3.8E-04 |
| Pbld2 | phenazine biosynthesis-like protein domain containing 2 | -3.0 | 1.0E-03 |
| Tmem144 | transmembrane protein 144 | -3.0 | 2.3E-04 |
| Slc5a11 | solute carrier family 5 (sodium/glucose cotransporter), member 11 | -3.0 | 2.6E-03 |
| Tmem140 | transmembrane protein 140 | -3.0 | 4.5E-04 |
| Nudt4 | nudix (nucleoside diphosphate linked moiety X)-type motif 4 | -3.0 | 1.6E-03 |
| Prm1 | protamine 1 | -3.0 | 9.0E-04 |
| Hes2 | hairy and enhancer of split 2 (Drosophila) | -3.0 | 1.1E-03 |
| Gcnt1 | glucosaminyl (N-acetyl) transferase 1, core 2 | -3.0 | 1.8E-04 |
| Dab1 | disabled homolog 1 (Drosophila) | -3.0 | 2.9E-04 |
| Slc6a20b | solute carrier family 6 (neurotransmitter transporter), member 20B | -3.0 | 3.5E-04 |
| Rnf152 | ring finger protein 152 | -3.0 | 5.0E-04 |
| Cyp4b1 | cytochrome P450, family 4, subfamily b, polypeptide 1 | -2.9 | 2.0E-04 |
| Nkg7 | natural killer cell group 7 sequence | -2.9 | 2.9E-04 |
| 4933405D12Rik | RIKEN cDNA 4933405D12 gene | -2.9 | 2.3E-04 |
| Rab30 | RAB30, member RAS oncogene family | -2.9 | 4.0E-04 |
| Pbld1 | phenazine biosynthesis-like protein domain containing 1 | -2.9 | 3.3E-04 |
| Xpnpep2 | X-prolyl aminopeptidase (aminopeptidase P) 2, membrane-bound | -2.9 | 1.0E-03 |
| 2010106E10Rik | RIKEN cDNA 2010106E10 gene | -2.9 | 1.5E-03 |
| Cyp2c29 | cytochrome P450, family 2, subfamily c, polypeptide 29 | -2.9 | 2.8E-04 |
| Smcp | sperm mitochondria-associated cysteine-rich protein | -2.9 | 6.4E-04 |
| Klrd1 | killer cell lectin-like receptor, subfamily D, member 1 | -2.9 | 2.4E-04 |
| Hpgd | hydroxyprostaglandin dehydrogenase 15 (NAD) | -2.9 | 2.8E-04 |
| Chst8 | carbohydrate (N-acetylgalactosamine 4-0) sulfotransferase 8 | -2.9 | 1.7E-03 |
| Tmem86a | transmembrane protein 86A | -2.8 | 5.1E-03 |
| Cd8a | CD8 antigen, alpha chain | -2.8 | 6.9E-04 |
| Cyp2c37 | cytochrome P450, family 2. subfamily c, polypeptide 37 | -2.8 | 2.7E-04 |

| | | | |
|---|---|---|---|
| Gm5483 | predicted gene 5483 | -2.8 | 2.3E-04 |
| Slc16a10 | solute carrier family 16 (monocarboxylic acid transporters), member 10 | -2.8 | 1.7E-03 |
| Wdr65 | WD repeat domain 65 | -2.8 | 4.3E-04 |
| Slc25a48 | solute carrier family 25, member 48 | -2.8 | 6.4E-03 |
| Cyp2c67 | cytochrome P450, family 2, subfamily c, polypeptide 67 | -2.8 | 4.2E-04 |
| Mylk3 | myosin light chain kinase 3 | -2.8 | 7.9E-04 |
| Mreg | melanoregulin | -2.8 | 8.4E-04 |
| Gm4055 | predicted gene 4055 | -2.8 | 7.6E-04 |
| Cndp1 | carnosine dipeptidase 1 (metallopeptidase M20 family) | -2.8 | 3.2E-04 |
| Snta1 | syntrophin, acidic 1 | -2.8 | 2.5E-04 |
| AI646023 | expressed sequence AI646023 | -2.7 | 6.6E-04 |
| A730089K16Rik | RIKEN cDNA A730089K16 gene | -2.7 | 3.0E-04 |
| Irf7 | interferon regulatory factor 7 | -2.7 | 3.4E-04 |
| Aqp1 | aquaporin 1 | -2.7 | 4.3E-04 |
| Gsta4 | glutathione S-transferase, alpha 4 | -2.7 | 5.6E-04 |
| Hsd3b6 | hydroxy-delta-5-steroid dehydrogenase, 3 beta- and steroid delta-isomerase 6 | -2.7 | 4.5E-04 |
| Faah | fatty acid amide hydrolase | -2.7 | 2.8E-04 |
| Treh | trehalase (brush-border membrane glycoprotein) | -2.7 | 2.8E-03 |
| Gm7967 | predicted gene 7967 | -2.7 | 4.1E-04 |
| Odf3b | outer dense fiber of sperm tails 3B | -2.7 | 2.3E-03 |
| Cyp2c39 | cytochrome P450, family 2, subfamily c, polypeptide 39 | -2.7 | 3.2E-04 |
| Cyp2c68 | cytochrome P450, family 2, subfamily c, polypeptide 68 | -2.6 | 5.3E-04 |
| Fam118a | family with sequence similarity 118, member A | -2.6 | 1.8E-03 |
| Adck3 | aarF domain containing kinase 3 | -2.6 | 4.4E-04 |
| Ugt2a3 | UDP glucuronosyltransferase 2 family, polypeptide A3 | -2.6 | 4.3E-04 |
| H2-Aa | histocompatibility 2, class II antigen A, alpha | -2.6 | 3.9E-04 |
| Ifit3 | interferon-induced protein with tetratricopeptide repeats 3 | -2.6 | 3.6E-03 |
| Slc7a15 | solute carrier family 7 (cationic amino acid transporter, y+ system), member 15 | -2.6 | 1.0E-03 |
| Neu1 | neuraminidase 1 | -2.6 | 4.4E-04 |
| BC026762 | cDNA sequence BC026762 | -2.6 | 5.1E-04 |
| Gm4316 | predicted gene 4316 | -2.6 | 2.3E-03 |
| Sema6a | sema domain, transmembrane domain (TM), and cytoplasmic domain, (semaphorin) 6A | -2.6 | 1.6E-03 |
| Bglap-rs1 | bone gamma-carboxyglutamate protein, related sequence 1 | -2.6 | 1.1E-03 |
| Maob | monoamine oxidase B | -2.6 | 2.2E-03 |
| Nr2e3 | nuclear receptor subfamily 2, group E, member 3 | -2.6 | 3.7E-04 |
| 2010002M12Rik | RIKEN cDNA 2010002M12 gene | -2.6 | 9.9E-04 |
| Hsd17b2 | hydroxysteroid (17-beta) dehydrogenase 2 | -2.5 | 3.2E-04 |
| 4933405E24Rik | RIKEN cDNA 4933405E24 gene | -2.5 | 1.8E-03 |
| Mettl7b | methyltransferase like 7B | -2.5 | 6.7E-04 |

| | | | |
|---|---|---|---|
| Serpina1f | serine (or cysteine) peptidase inhibitor, clade A, member 1F | -2.5 | 5.6E-04 |
| Il22ra2 | interleukin 22 receptor, alpha 2 | -2.5 | 9.7E-04 |
| Tox4 | TOX high mobility group box family member 4 | -2.5 | 1.5E-03 |
| Rasd2 | RASD family, member 2 | -2.5 | 1.4E-03 |
| Slc12a5 | solute carrier family 12, member 5 | -2.5 | 1.7E-03 |
| Acox2 | acyl-Coenzyme A oxidase 2, branched chain | -2.5 | 3.3E-04 |
| C81189 | expressed sequence C81189 | -2.5 | 3.8E-04 |
| LOC100039675 | uncharacterized LOC100039675 | -2.5 | 2.4E-03 |
| Hs3st6 | heparan sulfate (glucosamine) 3-O-sulfotransferase 6 | -2.5 | 2.3E-03 |
| Cpm | carboxypeptidase M | -2.5 | 1.5E-03 |
| Dclk3 | doublecortin-like kinase 3 | -2.5 | 4.2E-04 |
| BC089491 | cDNA sequence BC089491 | -2.5 | 5.5E-03 |
| Tnfaip8l2 | tumor necrosis factor, alpha-induced protein 8-like 2 | -2.5 | 2.0E-03 |
| Naaladl1 | N-acetylated alpha-linked acidic dipeptidase-like 1 | -2.5 | 2.8E-03 |
| Amy1 | amylase 1, salivary | -2.5 | 7.3E-03 |
| Rnf167 | ring finger protein 167 | -2.5 | 3.8E-03 |
| Bmp1 | bone morphogenetic protein 1 | -2.4 | 1.3E-03 |
| Bglap2 | bone gamma-carboxyglutamate protein 2 | -2.4 | 1.6E-03 |
| Olfr90 | olfactory receptor 90 | -2.4 | 7.6E-04 |
| Oasl2 | 2'-5' oligoadenylate synthetase-like 2 | -2.4 | 6.4E-04 |
| Vwa1 | von Willebrand factor A domain containing 1 | -2.4 | 2.0E-03 |
| 4930539E08Rik | RIKEN cDNA 4930539E08 gene | -2.4 | 6.1E-03 |
| Vnn1 | vanin 1 | -2.4 | 1.1E-03 |
| Sytl3 | synaptotagmin-like 3 | -2.4 | 8.8E-04 |
| Slc17a4 | solute carrier family 17 (sodium phosphate), member 4 | -2.4 | 1.0E-03 |
| Gsta3 | glutathione S-transferase, alpha 3 | -2.4 | 2.7E-03 |
| Tnfsf10 | tumor necrosis factor (ligand) superfamily, member 10 | -2.4 | 8.6E-04 |
| Hoga1 | 4-hydroxy-2-oxoglutarate aldolase 1 | -2.4 | 1.7E-03 |
| Gm10732 | predicted gene 10732 | -2.4 | 6.6E-04 |
| Akr1c19 | aldo-keto reductase family 1, member C19 | -2.4 | 5.2E-03 |
| Lpar3 | lysophosphatidic acid receptor 3 | -2.4 | 5.3E-04 |
| Gm14085 | predicted gene 14085 | -2.4 | 8.0E-04 |
| Ankrd29 | ankyrin repeat domain 29 | -2.4 | 7.1E-04 |
| Mep1a | meprin 1 alpha | -2.4 | 2.3E-03 |
| Abcc6 | ATP-binding cassette, sub-family C (CFTR/MRP), member 6 | -2.4 | 1.1E-03 |
| Proz | protein Z, vitamin K-dependent plasma glycoprotein | -2.4 | 1.0E-03 |
| Tgm5 | transglutaminase 5 | -2.4 | 4.5E-04 |
| Akr1c14 | aldo-keto reductase family 1, member C14 | -2.4 | 5.1E-04 |
| Ppp4r4 | protein phosphatase 4, regulatory subunit 4 | -2.4 | 4.2E-04 |
| A930038B10Rik | RIKEN cDNA A930038B10 gene | -2.4 | 4.4E-04 |
| Adra2c | adrenergic receptor, alpha 2c | -2.4 | 4.2E-03 |
| Mgst1 | microsomal glutathione S-transferase 1 | -2.4 | 5.6E-04 |
| Gzmk | granzyme K | -2.3 | 2.4E-03 |
| Wipf3 | WAS/WASL interacting protein family, member 3 | -2.3 | 4.4E-04 |
| Pmaip1 | phorbol-12-myristate-13-acetate-induced protein 1 | -2.3 | 1.8E-03 |
| Upk1b | uroplakin 1B | -2.3 | 5.6E-04 |
| 2310004I24Rik | RIKEN cDNA 2310004I24 gene | -2.3 | 5.1E-04 |

| | | | |
|---|---|---|---|
| Car12 | carbonic anyhydrase 12 | -2.3 | 1.4E-03 |
| Enpep | glutamyl aminopeptidase | -2.3 | 1.3E-03 |
| Mpst | mercaptopyruvate sulfurtransferase | -2.3 | 5.0E-04 |
| Stfa2l1 | stefin A2 like 1 | -2.3 | 9.1E-04 |
| Gm8909 | predicted gene 8909 | -2.3 | 4.6E-03 |
| Gpr172b | G protein-coupled receptor 172B | -2.3 | 8.8E-04 |
| Ttr | transthyretin | -2.3 | 5.0E-04 |
| Ttll2 | tubulin tyrosine ligase-like family, member 2 | -2.3 | 2.5E-03 |
| Cd3g | CD3 antigen, gamma polypeptide | -2.3 | 1.5E-03 |
| Mex3b | mex3 homolog B (C. elegans) | -2.3 | 1.7E-03 |
| Apol7a | apolipoprotein L 7a | -2.3 | 1.6E-03 |
| Gm5602 | predicted gene 5602 | -2.3 | 1.0E-03 |
| Rdh16 | retinol dehydrogenase 16 | -2.3 | 1.7E-03 |
| Ceacam20 | carcinoembryonic antigen-related cell adhesion molecule 20 | -2.3 | 6.7E-04 |
| Baat | bile acid-Coenzyme A: amino acid N-acyltransferase | -2.3 | 7.0E-04 |
| Gm5431 | predicted gene 5431 | -2.3 | 1.3E-03 |
| Abcg8 | ATP-binding cassette, sub-family G (WHITE), member 8 | -2.3 | 6.2E-04 |
| Lama3 | laminin, alpha 3 | -2.3 | 2.0E-03 |
| 4930428E23Rik | RIKEN cDNA 4930428E23 gene | -2.3 | 1.7E-03 |
| Anxa13 | annexin A13 | -2.3 | 6.9E-04 |
| Wfdc2 | WAP four-disulfide core domain 2 | -2.3 | 8.4E-04 |
| Hexb | hexosaminidase B | -2.3 | 1.1E-03 |
| Lats2 | large tumor suppressor 2 | -2.3 | 6.7E-04 |
| Dpyd | dihydropyrimidine dehydrogenase | -2.3 | 5.7E-04 |
| Zmat4 | zinc finger, matrin type 4 | -2.2 | 4.0E-03 |
| LOC633417 | h-2 class I histocompatibility antigen, TLA(B) alpha chain-like | -2.2 | 6.0E-04 |
| Nts | neurotensin | -2.2 | 3.9E-03 |
| Eif2c4 | eukaryotic translation initiation factor 2C, 4 | -2.2 | 1.4E-03 |
| Reg3d | regenerating islet-derived 3 delta | -2.2 | 7.6E-03 |
| Inmt | indolethylamine N-methyltransferase | -2.2 | 5.8E-03 |
| Nr1i3 | nuclear receptor subfamily 1, group I, member 3 | -2.2 | 7.2E-04 |
| Lect2 | leukocyte cell-derived chemotaxin 2 | -2.2 | 7.3E-03 |
| Cxcl9 | chemokine (C-X-C motif) ligand 9 | -2.2 | 7.0E-03 |
| Slc41a3 | solute carrier family 41, member 3 | -2.2 | 3.2E-03 |
| Mt1 | metallothionein 1 | -2.2 | 2.0E-03 |
| Fam151a | family with sequence simliarity 151, member A | -2.2 | 2.5E-03 |
| Cd4 | CD4 antigen | -2.2 | 1.2E-03 |
| Cndp2 | CNDP dipeptidase 2 (metallopeptidase M20 family) | -2.2 | 6.7E-04 |
| Hbb-b1 | hemoglobin, beta adult major chain | -2.2 | 5.5E-03 |
| Tfpi | tissue factor pathway inhibitor | -2.2 | 1.1E-03 |
| 9130016M20Rik | RIKEN cDNA 9130016M20 gene | -2.2 | 2.8E-03 |
| 4930420K17Rik | RIKEN cDNA 4930420K17 gene | -2.2 | 1.5E-03 |
| Hist1h1c | histone cluster 1, H1c | -2.2 | 7.2E-04 |
| Gm4402 | predicted gene 4402 | -2.2 | 3.0E-03 |
| Fasl | Fas ligand (TNF superfamily, member 6) | -2.2 | 1.0E-03 |
| Beta-s | hemoglobin subunit beta-1-like | -2.2 | 5.1E-03 |

| | | | |
|---|---|---|---|
| Gm11127 | predicted gene 11127 | -2.2 | 1.4E-03 |
| Slc27a2 | solute carrier family 27 (fatty acid transporter), member 2 | -2.2 | 6.6E-04 |
| 2310007B03Rik | RIKEN cDNA 2310007B03 gene | -2.2 | 8.0E-04 |
| Krba1 | KRAB-A domain containing 1 | -2.2 | 1.2E-03 |
| Oat | ornithine aminotransferase | -2.2 | 8.4E-04 |
| Dio1 | deiodinase, iodothyronine, type I | -2.2 | 1.8E-03 |
| Gab1 | growth factor receptor bound protein 2-associated protein 1 | -2.2 | 1.3E-03 |
| Hbb-b2 | hemoglobin, beta adult minor chain | -2.2 | 1.8E-03 |
| C87977 | expressed sequence C87977 | -2.2 | 7.2E-04 |
| Aplp1 | amyloid beta (A4) precursor-like protein 1 | -2.2 | 9.8E-04 |
| Bex1 | brain expressed gene 1 | -2.2 | 1.5E-03 |
| Pepd | peptidase D | -2.2 | 2.0E-03 |
| Rab19 | RAB19, member RAS oncogene family | -2.2 | 4.2E-03 |
| H2-T18 | histocompatibility 2, T region locus 18 | -2.2 | 1.2E-03 |
| Slc44a1 | solute carrier family 44, member 1 | -2.2 | 7.4E-04 |
| Optn | optineurin | -2.2 | 7.6E-04 |
| 4930458D05Rik | RIKEN cDNA 4930458D05 gene | -2.2 | 2.5E-03 |
| Bcmo1 | beta-carotene 15,15'-monooxygenase | -2.2 | 4.3E-03 |
| Cyp4f17 | cytochrome P450, family 4, subfamily f, polypeptide 17 | -2.2 | 6.9E-04 |
| Gm7854 | predicted gene 7854 | -2.2 | 1.1E-03 |
| Fmo4 | flavin containing monooxygenase 4 | -2.2 | 6.6E-04 |
| Nxf7 | nuclear RNA export factor 7 | -2.2 | 3.1E-03 |
| 9430038I01Rik | RIKEN cDNA 9430038I01 gene | -2.2 | 3.9E-03 |
| Klkb1 | kallikrein B, plasma 1 | -2.1 | 4.6E-03 |
| Tst | thiosulfate sulfurtransferase, mitochondrial | -2.1 | 7.9E-04 |
| 4930558N11Rik | RIKEN cDNA 4930558N11 gene | -2.1 | 1.4E-03 |
| Naip7 | NLR family, apoptosis inhibitory protein 7 | -2.1 | 1.0E-03 |
| Cyp3a13 | cytochrome P450, family 3, subfamily a, polypeptide 13 | -2.1 | 6.9E-04 |
| Plg | plasminogen | -2.1 | 4.7E-03 |
| Fam132a | family with sequence similarity 132, member A | -2.1 | 1.8E-03 |
| Slc22a4 | solute carrier family 22 (organic cation transporter), member 4 | -2.1 | 1.6E-03 |
| Sfrp5 | secreted frizzled-related sequence protein 5 | -2.1 | 1.1E-03 |
| Me3 | malic enzyme 3, NADP(+)-dependent, mitochondrial | -2.1 | 6.4E-03 |
| Rdh9 | retinol dehydrogenase 9 | -2.1 | 1.3E-03 |
| Sowaha | sosondowah ankyrin repeat domain family member A | -2.1 | 2.8E-03 |
| Pcdh19 | protocadherin 19 | -2.1 | 9.7E-04 |
| Sstr3 | somatostatin receptor 3 | -2.1 | 8.9E-03 |
| Fbxw17 | F-box and WD-40 domain protein 17 | -2.1 | 9.9E-04 |
| Clic5 | chloride intracellular channel 5 | -2.1 | 7.6E-04 |
| Igtp | interferon gamma induced GTPase | -2.1 | 1.1E-03 |
| Atg16l2 | autophagy related 16 like 2 (S. cerevisiae) | -2.1 | 7.6E-04 |
| H2-T3 | histocompatibility 2, T region locus 3 | -2.1 | 3.1E-03 |
| Trim30e-ps1 | tripartite motif-containing 30E, pseudogene 1 | -2.1 | 1.1E-03 |

| | | | |
|---|---|---|---|
| B4galt4 | UDP-Gal:betaGlcNAc beta 1,4-galactosyltransferase, polypeptide 4 | -2.1 | 1.5E-03 |
| Cd36 | CD36 antigen | -2.1 | 1.1E-03 |
| H2-DMa | histocompatibility 2, class II, locus DMa | -2.1 | 2.8E-03 |
| Hist1h2ba | histone cluster 1, H2ba | -2.1 | 8.0E-04 |
| Cplx2 | complexin 2 | -2.1 | 9.9E-04 |
| AW111846 | expressed sequence AW111846 | -2.1 | 8.2E-04 |
| Fbxo25 | F-box protein 25 | -2.1 | 1.4E-03 |
| Gm10872 | predicted gene 10872 | -2.1 | 1.4E-03 |
| Rnf180 | ring finger protein 180 | -2.1 | 1.2E-03 |
| Skap1 | src family associated phosphoprotein 1 | -2.1 | 5.1E-03 |
| Tmem86b | transmembrane protein 86B | -2.1 | 4.8E-03 |
| Gm2921 | predicted gene 2921 | -2.1 | 2.7E-03 |
| Ppap2a | phosphatidic acid phosphatase type 2A | -2.1 | 1.0E-03 |
| Acot1 | acyl-CoA thioesterase 1 | -2.1 | 9.6E-04 |
| Xcl1 | chemokine (C motif) ligand 1 | -2.1 | 1.8E-03 |
| Cyp3a41b | cytochrome P450, family 3, subfamily a, polypeptide 41B | -2.1 | 3.7E-03 |
| Cxcl10 | chemokine (C-X-C motif) ligand 10 | -2.1 | 4.9E-03 |
| Fbxo32 | F-box protein 32 | -2.1 | 1.9E-03 |
| Map2k7 | mitogen-activated protein kinase kinase 7 | -2.1 | 1.2E-03 |
| 9130024F11Rik | RIKEN cDNA 9130024F11 gene | -2.1 | 8.6E-04 |
| Slc23a1 | solute carrier family 23 (nucleobase transporters), member 1 | -2.1 | 8.2E-04 |
| C2 | complement component 2 (within H-2S) | -2.1 | 8.0E-04 |
| Plekhg6 | pleckstrin homology domain containing, family G (with RhoGef domain) member 6 | -2.1 | 2.0E-03 |
| H2-Ab1 | histocompatibility 2, class II antigen A, beta 1 | -2.1 | 1.5E-03 |
| Apoa1 | apolipoprotein A-I | -2.1 | 4.0E-03 |
| Adamtsl5 | ADAMTS-like 5 | -2.1 | 1.6E-03 |
| Arl13b | ADP-ribosylation factor-like 13B | -2.1 | 1.6E-03 |
| Trib3 | tribbles homolog 3 (Drosophila) | -2.1 | 1.5E-03 |
| Rnf13 | ring finger protein 13 | -2.1 | 1.1E-03 |
| Gprin3 | GPRIN family member 3 | -2.1 | 3.5E-03 |
| Id2 | inhibitor of DNA binding 2 | -2.1 | 2.2E-03 |
| Vav3 | vav 3 oncogene | -2.1 | 2.1E-03 |
| Ptprr | protein tyrosine phosphatase, receptor type, R | -2.1 | 1.2E-03 |
| Gm10532 | predicted gene 10532 | -2.0 | 6.7E-03 |
| Cyp2b9 | cytochrome P450, family 2, subfamily b, polypeptide 9 | -2.0 | 1.3E-03 |
| Hba-a1 | hemoglobin alpha, adult chain 1 | -2.0 | 8.8E-03 |
| Lrp12 | low density lipoprotein-related protein 12 | -2.0 | 2.1E-03 |
| Rorc | RAR-related orphan receptor gamma | -2.0 | 1.6E-03 |
| Cd96 | CD96 antigen | -2.0 | 2.2E-03 |
| Dmrt3 | doublesex and mab-3 related transcription factor 3 | -2.0 | 1.7E-03 |
| Crot | carnitine O-octanoyltransferase | -2.0 | 9.6E-04 |
| Cables1 | CDK5 and Abl enzyme substrate 1 | -2.0 | 1.0E-03 |
| Rab11fip3 | RAB11 family interacting protein 3 (class II) | -2.0 | 5.3E-03 |
| I830012O16Rik | RIKEN cDNA I830012O16 gene | -2.0 | 7.2E-03 |
| Btnl5 | butyrophilin-like 5 | -2.0 | 9.1E-04 |

| | | | |
|---|---|---|---|
| Slc25a36 | solute carrier family 25, member 36 | -2.0 | 2.4E-03 |
| Smad6 | MAD homolog 6 (Drosophila) | -2.0 | 1.7E-03 |
| Gm4203 | predicted gene 4203 | -2.0 | 1.2E-03 |
| Maoa | monoamine oxidase A | -2.0 | 2.4E-03 |
| Lrat | lecithin-retinol acyltransferase (phosphatidylcholine-retinol-O-acyltransferase) | -2.0 | 6.5E-03 |
| Efr3b | EFR3 homolog B (S. cerevisiae) | -2.0 | 3.3E-03 |
| Erbb3 | v-erb-b2 erythroblastic leukemia viral oncogene homolog 3 (avian) | -2.0 | 4.7E-03 |
| Sardh | sarcosine dehydrogenase | -2.0 | 2.5E-03 |
| Tmigd1 | transmembrane and immunoglobulin domain containing 1 | -2.0 | 9.9E-04 |
| Zfp385c | zinc finger protein 385C | -2.0 | 2.6E-03 |
| Def8 | differentially expressed in FDCP 8 | -2.0 | 3.1E-03 |
| Slc22a5 | solute carrier family 22 (organic cation transporter), member 5 | -2.0 | 1.0E-03 |
| Podn | podocan | -2.0 | 2.4E-03 |
| Dusp12 | dual specificity phosphatase 12 | -2.0 | 1.4E-03 |
| Ddit4l | DNA-damage-inducible transcript 4-like | -2.0 | 1.7E-03 |
| Plbd1 | phospholipase B domain containing 1 | -2.0 | 3.3E-03 |

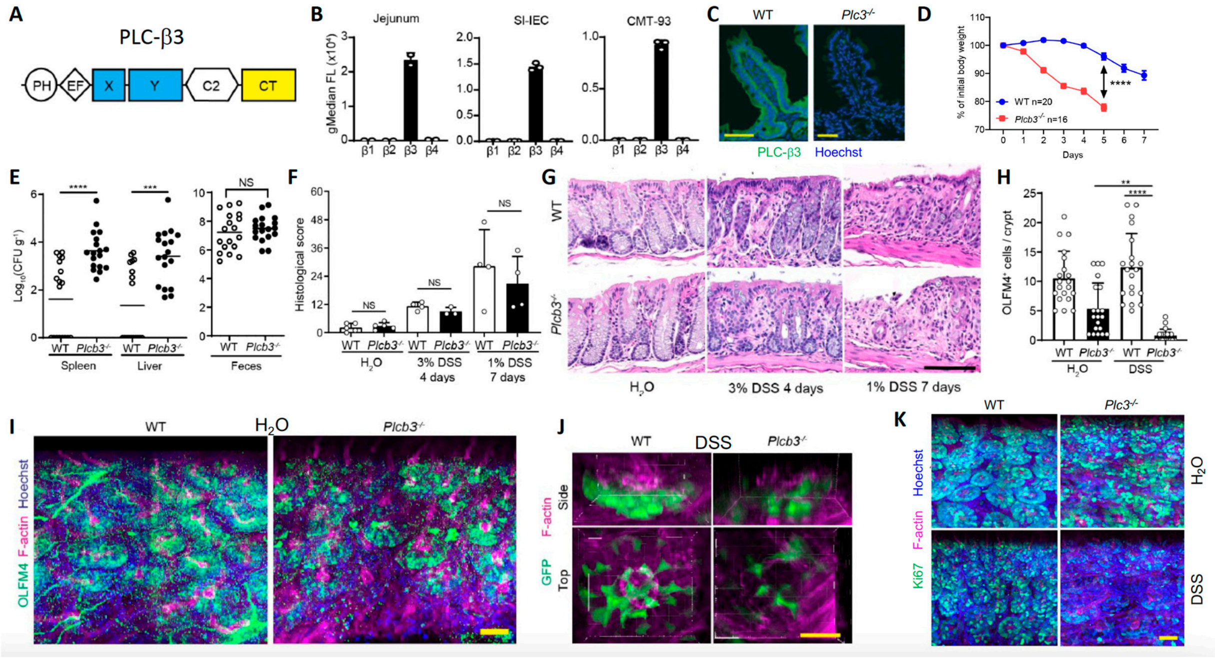

**Figure S1. Expression of PLC-β3 in IECs and phenotypic abnormalities in *Plcb3*$^{-/-}$ mice.**
(A) The domain structure of PLC-β3. PH, pleckstrin homology domain; EF, EF-hand domains; X and Y, split catalytic domain; C2, C2 domain; CT, C-terminal domain. (B) mRNA expression of PLC-β isoforms (β1-β4) in whole jejunum, isolated mouse small intestinal IECs, and CMT-93 cells was analyzed by DNA microarray. gMedian FL; geometric median fluorescence. Mean ± SD. (C) Confocal microscopy analysis of PLC-β3 (Green) in jejuna of WT and *Plcb3*$^{-/-}$ mice. Scale bar, 50 µm. (D) Changes in body weight of mice upon exposure to 3% DSS. (E) Live *Citrobacter rodentium* was orally injected to WT or *Plcb3*$^{-/-}$ mice. 6 days later, colony-forming units (CFU) of *C. rodentium* in indicated organs were measured and normalized by tissue weight. The graph shows combined results of three experiments. ***, p<0.001; ****, p<0.0001 by Mann-Whitney test. (F,G) Histological score was assessed with H&E-stained colons at day 4 of 3% DSS treatment or day 7 of 1% DSS treatment in a blinded manner. Shown are combined results of two experiments. The graph shows mean ± SD. Kruskal-Wallis test and Dunn's multiple comparison test was used for statistical analysis. NS: not significant. (H, I) WT and *Plcb3*$^{-/-}$ mice with or without DSS treatment were analyzed by confocal microscopy. Whole mount ilea were stained by Hoechst (nuclei), anti-Olfm4 (ISCs), and phalloidin (F-actin). (H) 20 crypts from one mouse per condition were analyzed for Olfm4$^{+}$ cells. ****, p < 0.0001; **, p < 0.01 by Kruskal-Wallis test and Dunn's multiple comparison test. (I) Crypt cells under $H_2O$ conditions (compare to **Figure 1H**) are shown. Scale bar, 10 µm. (J) Whole mount ilea from *Plcb3*$^{-/-}$*;Lgr5-EGFP-IRES-CreERT2*$^{+}$ mice and control *Plcb3*$^{+/-}$*;Lgr5-EGFP-IRES-Cre-ERT2*$^{+}$ mice, which were treated with 3% DSS for 2 days, were observed by confocal microscopy. These images confirm the reduction of Olfm4$^{+}$Lgr5$^{+}$ ISCs in DSS-treated mice in **Figure 1H**. Scale bar, 10 µm. (K) Ki-67 (green) staining of whole mount ilea. Ki-67 staining was conducted once with whole mount tissue and twice with sections with similar results. Scale bar, 10 µm.

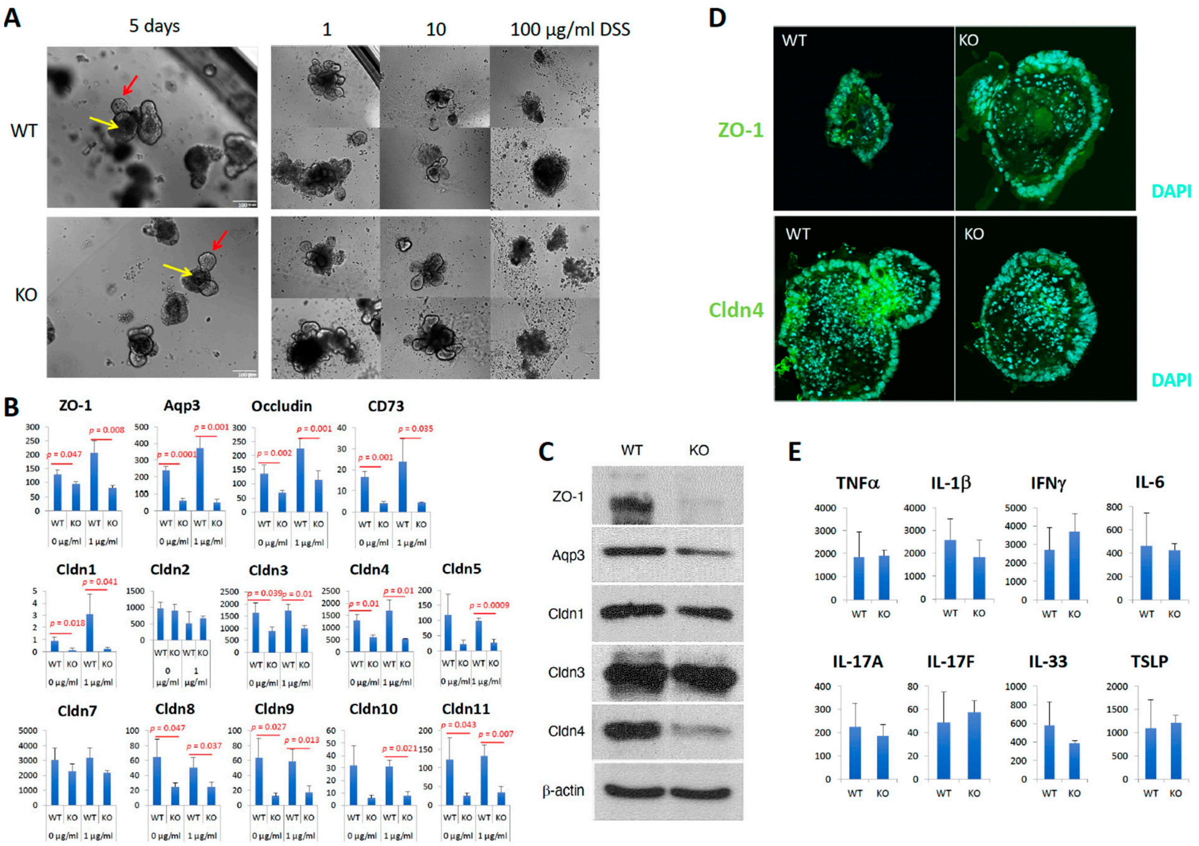


**Figure S2. Morphology and gene expression of crypt organoids and their responses to DSS exposure.** (A) Organoids were cultured from small intestinal crypts from WT and *Plcb3*$^{-/-}$ (KO) mice for 5 days (Left panels). Then, indicated concentrations of DSS were added to the cultures for 24 h (Right panels). Red arrows indicate the apical side of the epithelium, and yellow arrows indicate crypts. (B) RNA was isolated from crypt organoids cultured for 5 days under standard conditions and then exposed or not to 1 µg/ml of DSS for another day. qRT-PCR for tight junction proteins was performed. (C) Western blot analysis was done on crypt organoids cultured for 5 days under normal condition. Cldn, claudin. (D) Expression of ZO-1 and Claudin4 in 5-day crypt organoids was analyzed by immunofluorescent confocal microscopy. (E) RNA was isolated from 5-day crypt organoids and subjected to qRT-PCR.

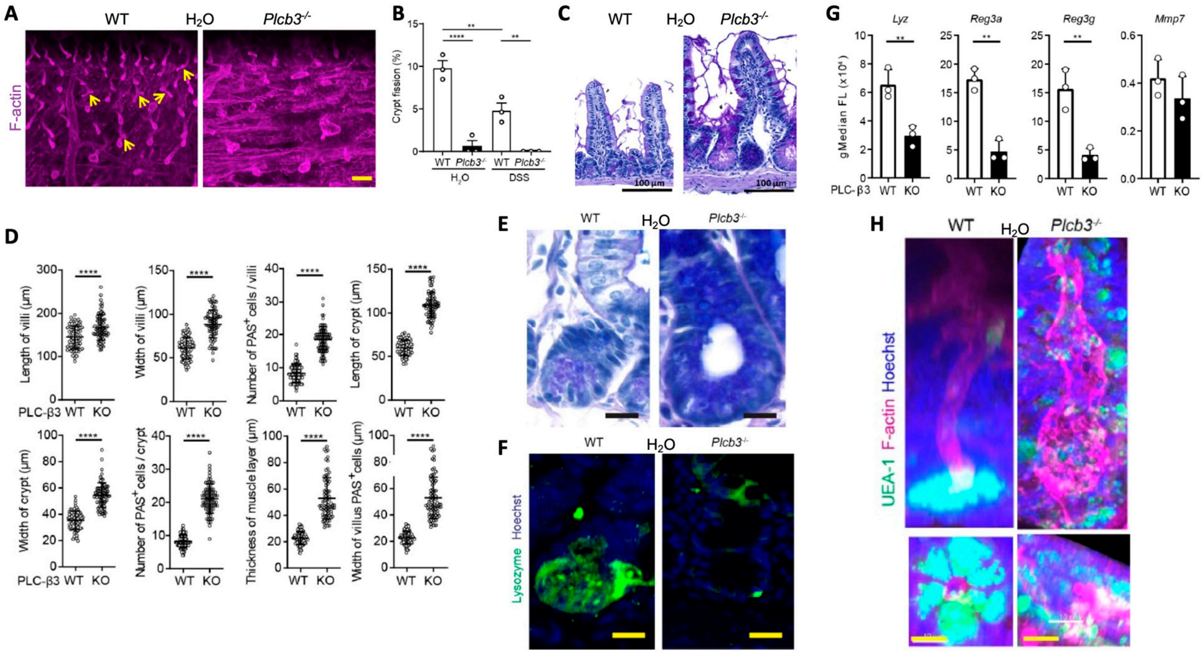

**Figure S3. Phenotypic abnormalities of small intestinal IECs of PLC-β3$^{-/-}$ mice.**
(A,B) F-actin channel of the images shown in Figure S1K was used to count crypt fissions. Arrows indicate crypt fissions. Scale bar, 10 µm. (B) Ratio of crypt fissions. Three different portions of the small intestine from the same mice were analyzed in each condition. ****, p < 0.0001; **, p < 0.01 by one way ANOVA and Holm-Sidak's multiple comparisons test. (C) PAS staining of ilea from WT and *Plcb3*$^{-/-}$ mice without DSS treatment. Scale bar, 100 µm. (D) Quantification of morphological abnormalities. 75 well-oriented crypt-villous structures from 3 different biological replicates were analyzed. ****, p<0.0001 by Mann-Whitney test. (E) Alcian blue staining of ilea from WT and *Plcb3*$^{-/-}$ mice without DSS treatment. Scale bar, 10 µm. (F) Lysozyme staining of ilea of mice without DSS treatment. Scale bar, 10 µm. (G) Microarray analysis of IECs from the small intestines. The reduced expression of *Lyz1* was confirmed by qRT-PCR. *, p<0.05; ****, p<0.0001 vs. WT by Student's t-test. (H) Whole mount ilea from mice without DSS treatment were stained by UEA 1 for α(1,2)fucose. Scale bar, 100 µm.

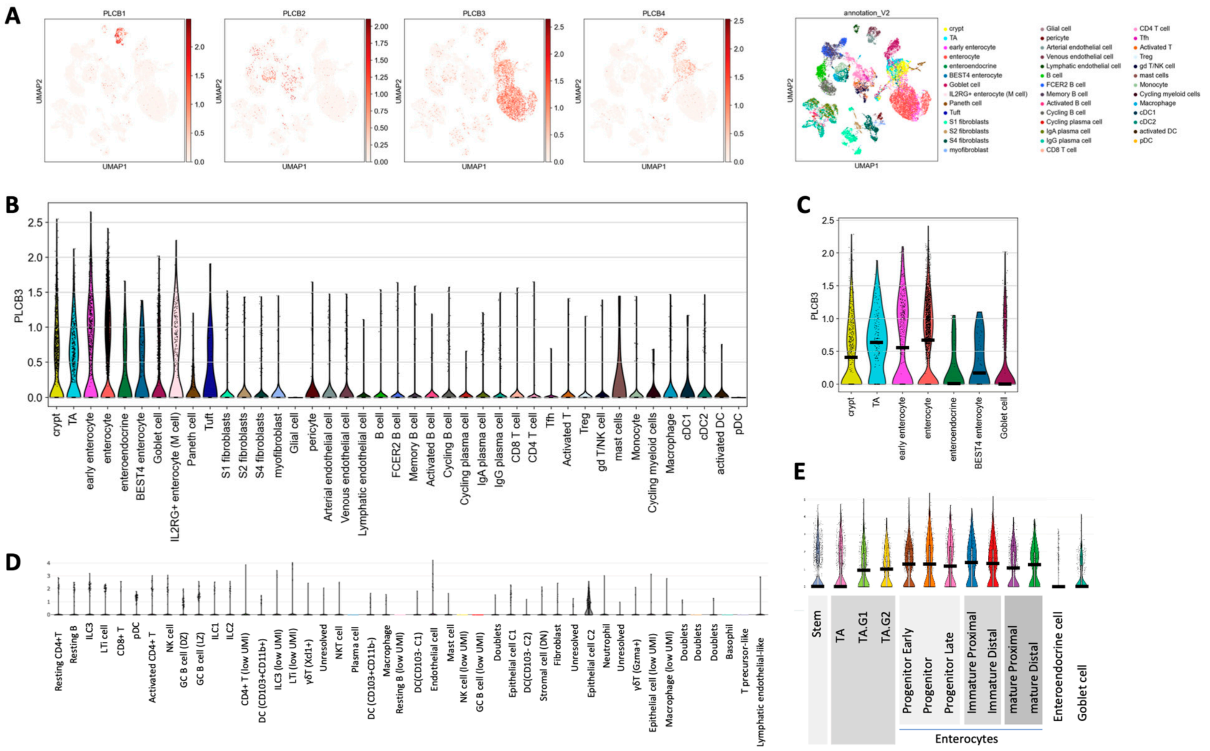


**Figure S4. Single-cell level mRNA expression of PLC-β isoforms in mice and humans.**
(A) UMAP projection of the expression levels of PLC-β isoforms in the publicly available single-cell RNA-seq data of pediatric ileal cells from healthy donors and CD patients (*1*). (B) Violin plots of *PLCB3* expression in each cell cluster in (A). (C) Violin plots of *PLCB3* expression during the epithelial cell development in healthy donor subset. (D) Violin plots of *Plcb3* expression in various cell types in mouse intestinal cells (*2*). UMI, unique molecular identifier; DN, double negative; C1, cluster 1; C2, cluster 2. (E) Violin plots of *Plcb3* expression in mouse small intestinal epithelial cells (*3*).

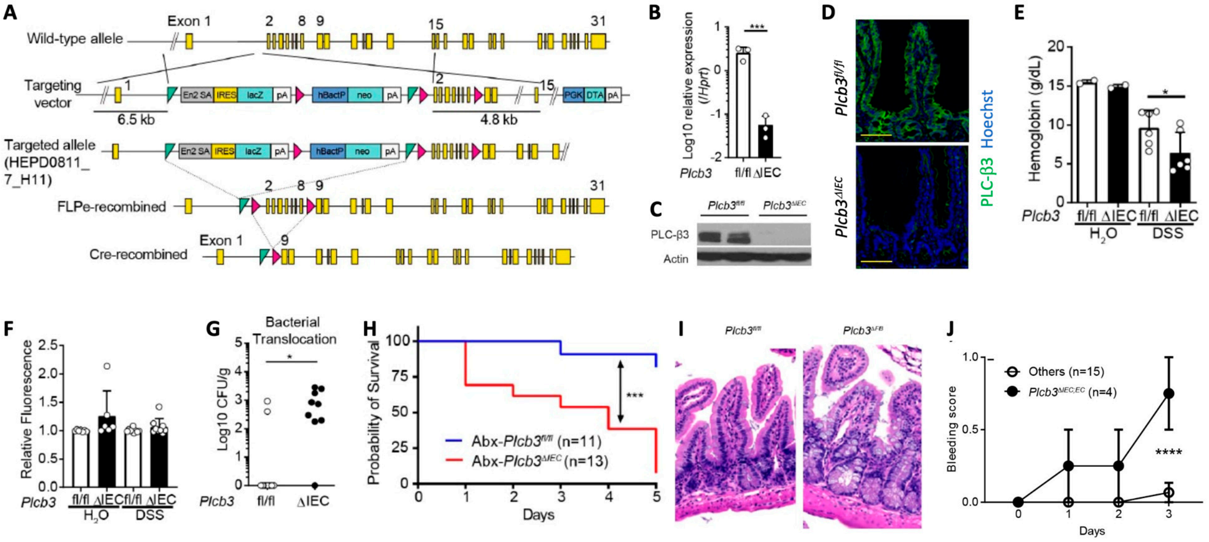


**Figure S5.** ***$Plcb3^{\Delta IEC}$*** **mice exhibit moderately increased DSS susceptibility.**
(A) Scheme for genetic manipulation to generate *Plcb3* cKO mice. (B-D) Deletion of PLC-β3 expression in small intestinal IECs of *$Plcb3^{\Delta IEC}$* mice by qRT-PCR (B) and by western blotting (C). ***, p<0.001 by two tailed Student's t-test. (D) Confocal microscopy analysis of PLC-β3 (green) in ilea of *$Plcb3^{fl/fl}$* and *$Plcb3^{\Delta IEC}$* mice. Scale bar, 50 µm. (E) Blood hemoglobin concentrations were measured after *$Plcb3^{fl/fl}$* or *$Plcb3^{\Delta IEC}$* mice were treated with 5% DSS or $H_2O$ for 7 days. *, p<0.05 by two tailed Student's t-test. (F,G) Intestinal permeability was measured by FITC-dextran (F) and *C. rodentium* (G) gavage. *, p<0.05 by Mann-Whitney test. (H) *$Plcb3^{fl/fl}$* and *$Plcb3^{\Delta IEC}$* mice were treated with antibiotics cocktail for 4 weeks, then, exposed to 3% DSS. The survival curve represents combined results of two experiments. ***, p<0.001, log-rank test. (I) HE staining of ilea from *$Plcb3^{fl/fl}$* and *$Plcb3^{\Delta FB}$* mice. (J) *$Plcb3^{\Delta IEC;EC}$* showed early intestinal bleeding following treatment with 3% DSS. ****, p<0.0001 by two-way ANOVA.

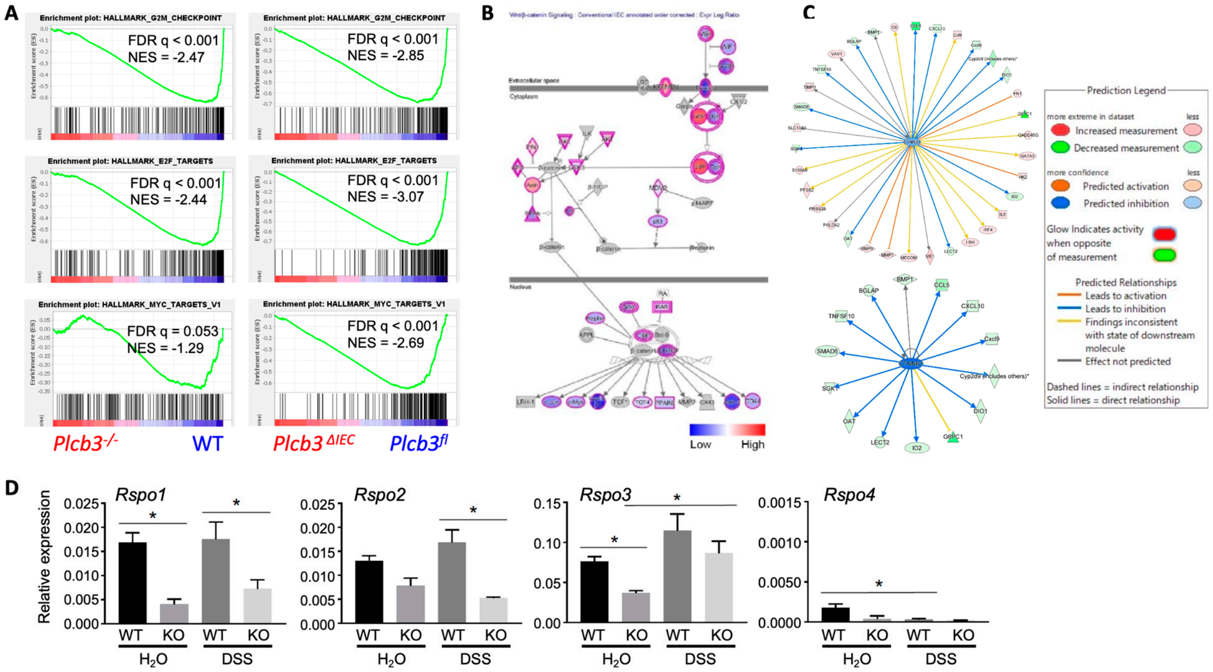


**Figure S6. PLC-β3 controls cell-cycle signature and Wnt/β-catenin signaling pathway genes in small intestinal IECs.**

(A) GSEA was performed on genes expressed by small intestinal IECs between *Plcb3*$^{-/-}$ and *Plcb3*$^{\Delta IEC}$ mice vs. their respective control mice for G2/M checkpoint genes, E2F target genes, and Myc target genes. (B,C) Ingenuity Pathway Analysis (IPA) on small intestinal IECs from *Plcb3*$^{-/-}$ and WT mice shows that PLC-β3 regulates the Wnt/β-catenin pathway (B). IPA also indicates up- or down-regulation of β-catenin-related down- or up-regulated genes, respectively, in *Plcb3*$^{-/-}$ and WT small intestinal IECs. (C). (D) Mesenchymal tissues were isolated from small intestines of WT and *Plcb3*$^{-/-}$ mice exposed to sterilized water or 3% DSS for 2 days. RNAs isolated were subjected to qRT-PCR analysis. *, p<0.05 by Student's t-test.

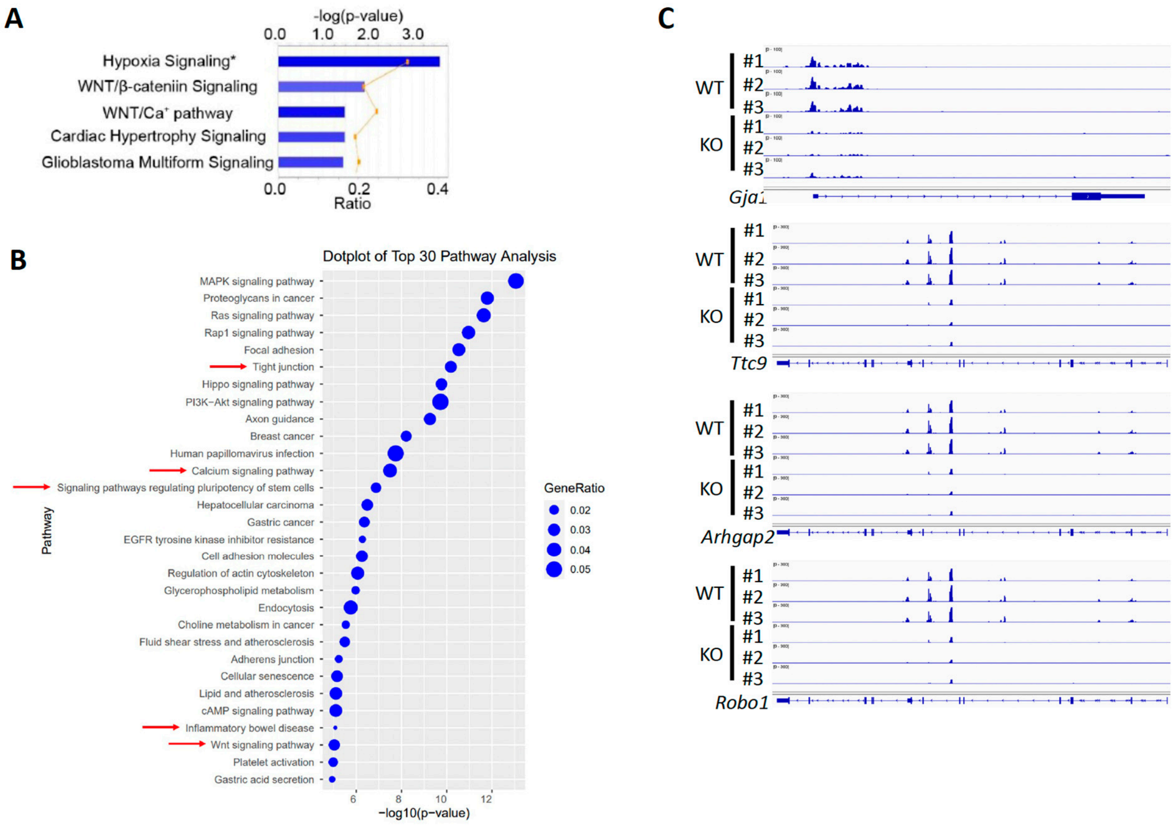


**Figure S7. PLC-β3 controls Wnt/β-catenin signaling in IECs at epigenetic and transcriptomic levels.** (A) Gene expression was analyzed in *Plcb3*-deficient and -sufficient CMT93 cells by Ingenuity Pathway Analysis (IPA). Top 5 signaling pathways most affected by *Plcb3* deficiency are shown. Bar charts and line graphs show the $-\log_{10}$(p value) and the ratios of genes included in each canonical pathway, respectively. *Hypoxia signaling in the Cardiovascular system is abbreviated. (B) Top 30 Pathway analysis of ATAC-seq data by clusterProfiler. Red arrows indicate pathways highly relevant to this study. (C) ATAC-seq analysis of *Plcb3*-deficient (KO) and -sufficient (WT) CMT-93 cells. Four gene loci potentially relevant to IEC functions and IBD pathogenesis are shown.

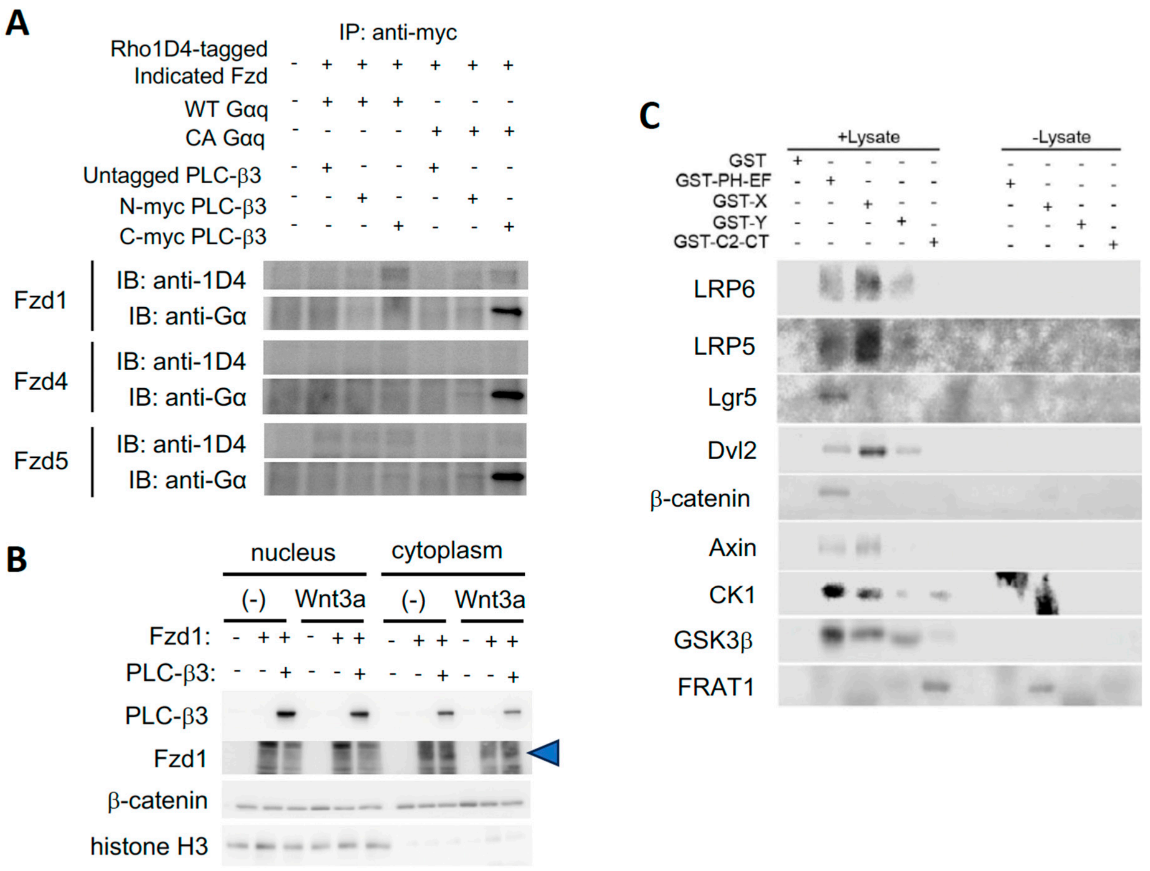


**Figure S8. PLC-β3 may control Wnt/β-catenin signaling in IECs by protein-protein interactions.** (A) Mammalian expression vectors with the indicated proteins as an insert and PLC-β3 constructs were co-transfected to HEK293T cells. WT or constitutively active (CA) mouse Gαq and Rho1D4-tagged different Fzd constructs were transfected as indicated. Anti-Myc antibody was used for immunoprecipitation. IP, immunoprecipitation; IB, immunoblotting. (B) HEK293T cells were transfected with Rho1D4-tagged Fzd1 expression vector with or without non-tagged PLC-β3 expression vector. The transfected cells were stimulated with Wnt3a and Afamin for 5 h, lysed, and fractionated into nuclear and cytoplasmic compartments. Expression of the indicated molecules were detected by western blotting. Arrowhead indicates Fzd1. (C) GST and GST-fusion proteins containing the indicated domains of mouse PLC-β3 cDNA were incubated with CMT-93 cell lysates (10 μg protein) or not. Glutathione agarose-bound proteins were analyzed by western blotting for the indicated proteins.

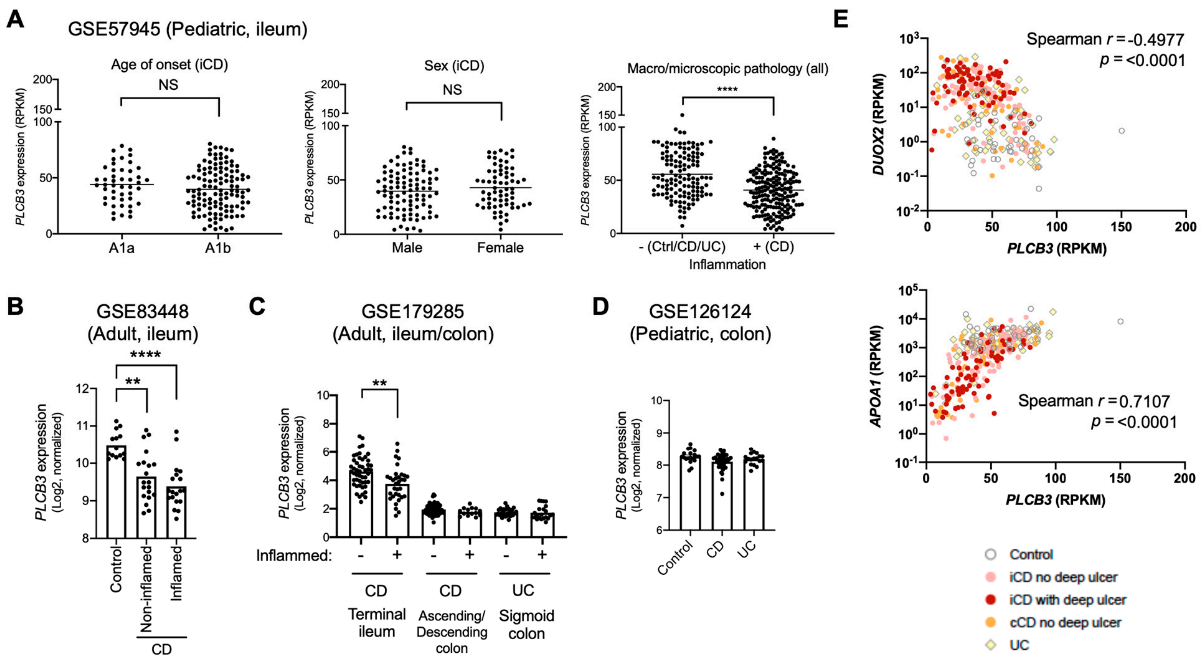


**Figure S9. Reduced expression of PLC-β3 in ileal biopsy specimens of CD patients.**
(A-D) Reduced *PLCB3* mRNA expression is shown in inflamed ieal CD samples, but not in other types of IBD, in the indicated datasets. (E) *PLCB3* expression is inversely correlated with *DUOX2* expression and positively correlated with *APOA1* expression in ileum. Ulcerated areas were avoided for biopsy even in the “with deep ulcer” samples.

# Uncropped western blot images

## Original scanned images for Figure 3E

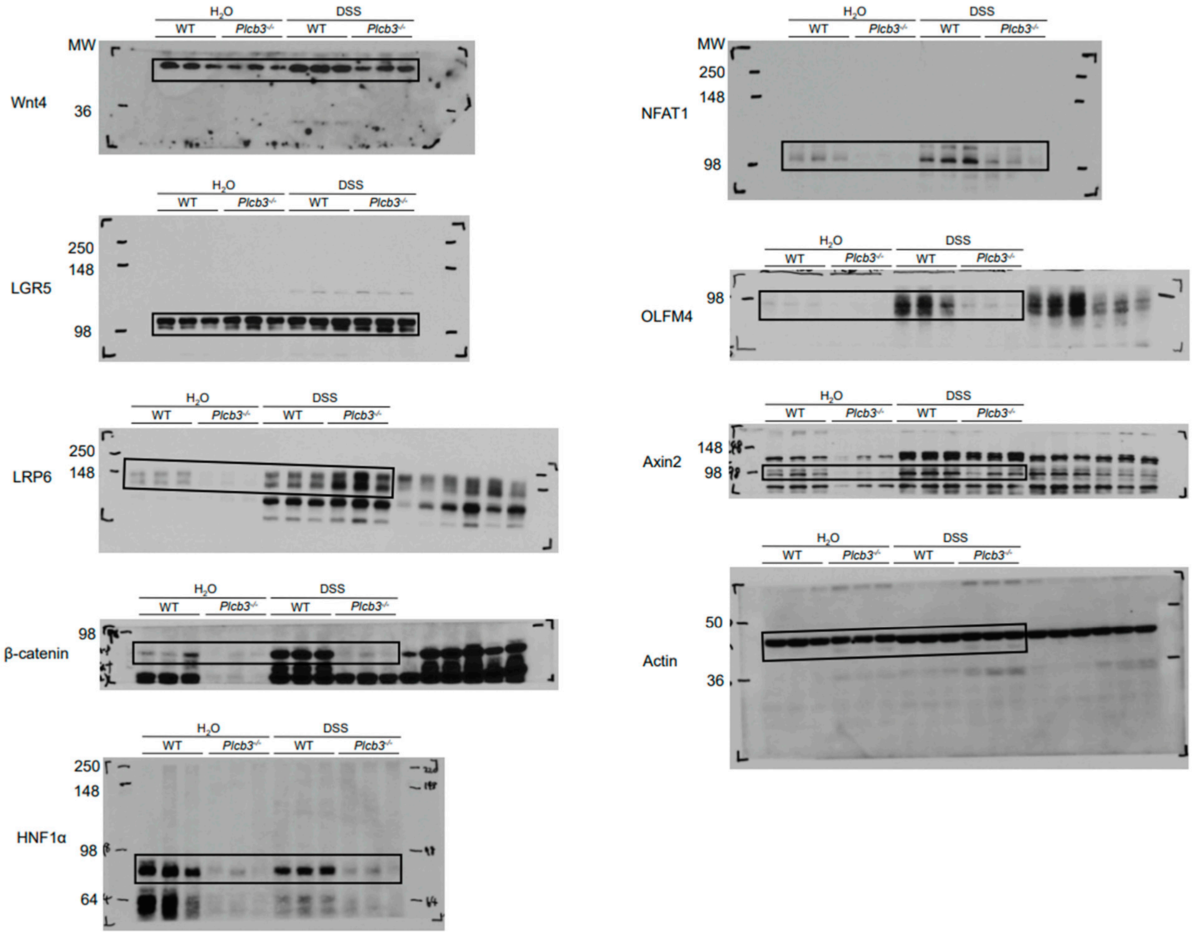

## Original scanned images for Figure 4A

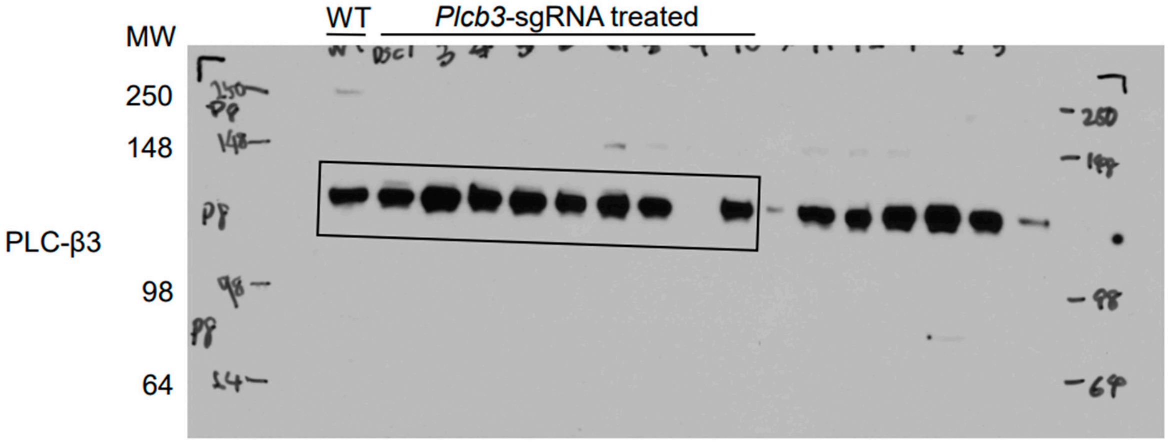


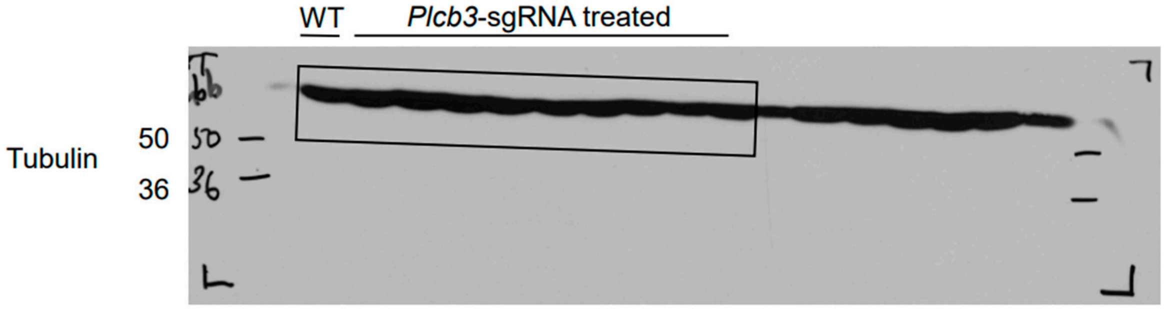

## Original scanned images for Figure4E

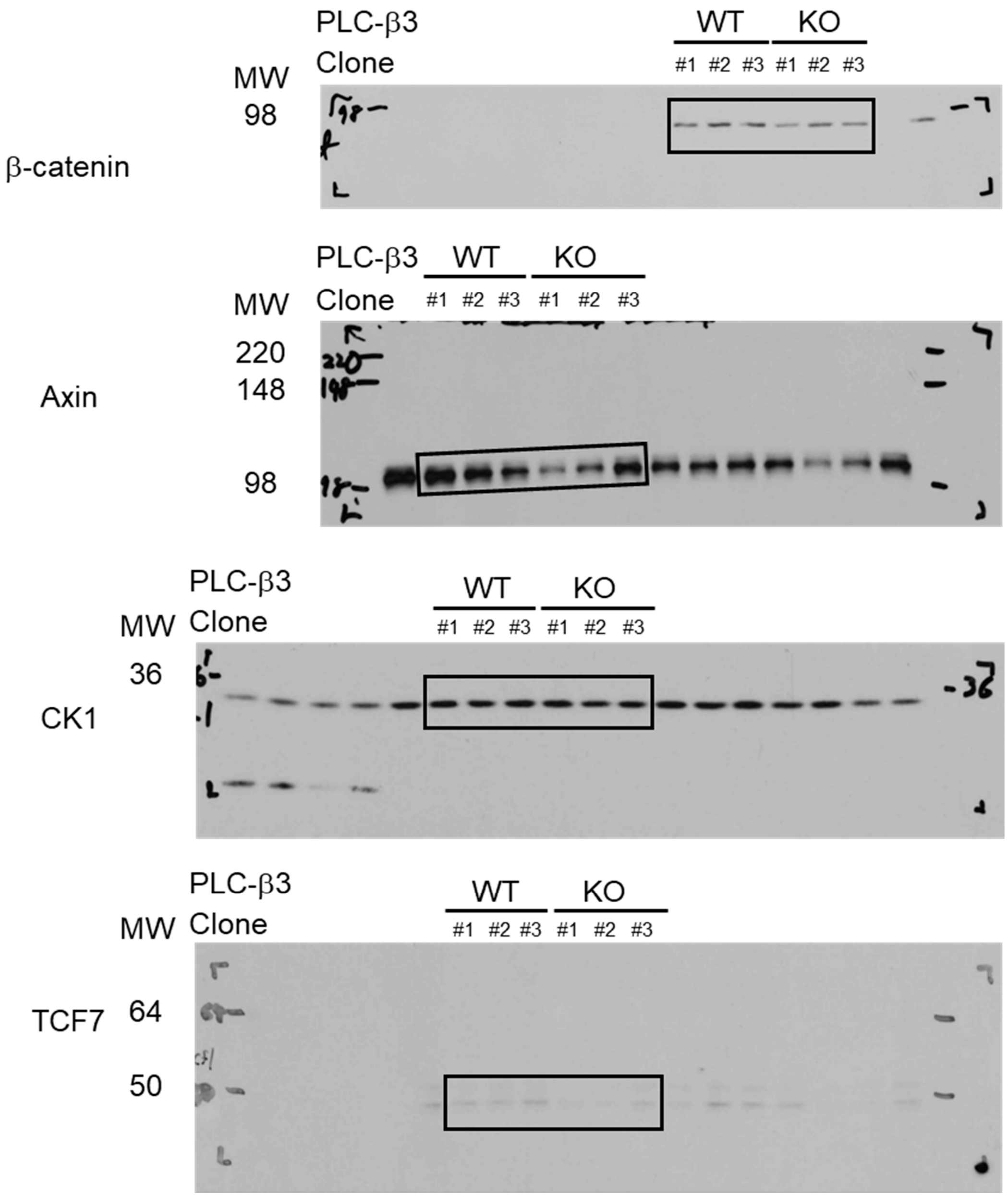

# Original scanned images for Figure 4H

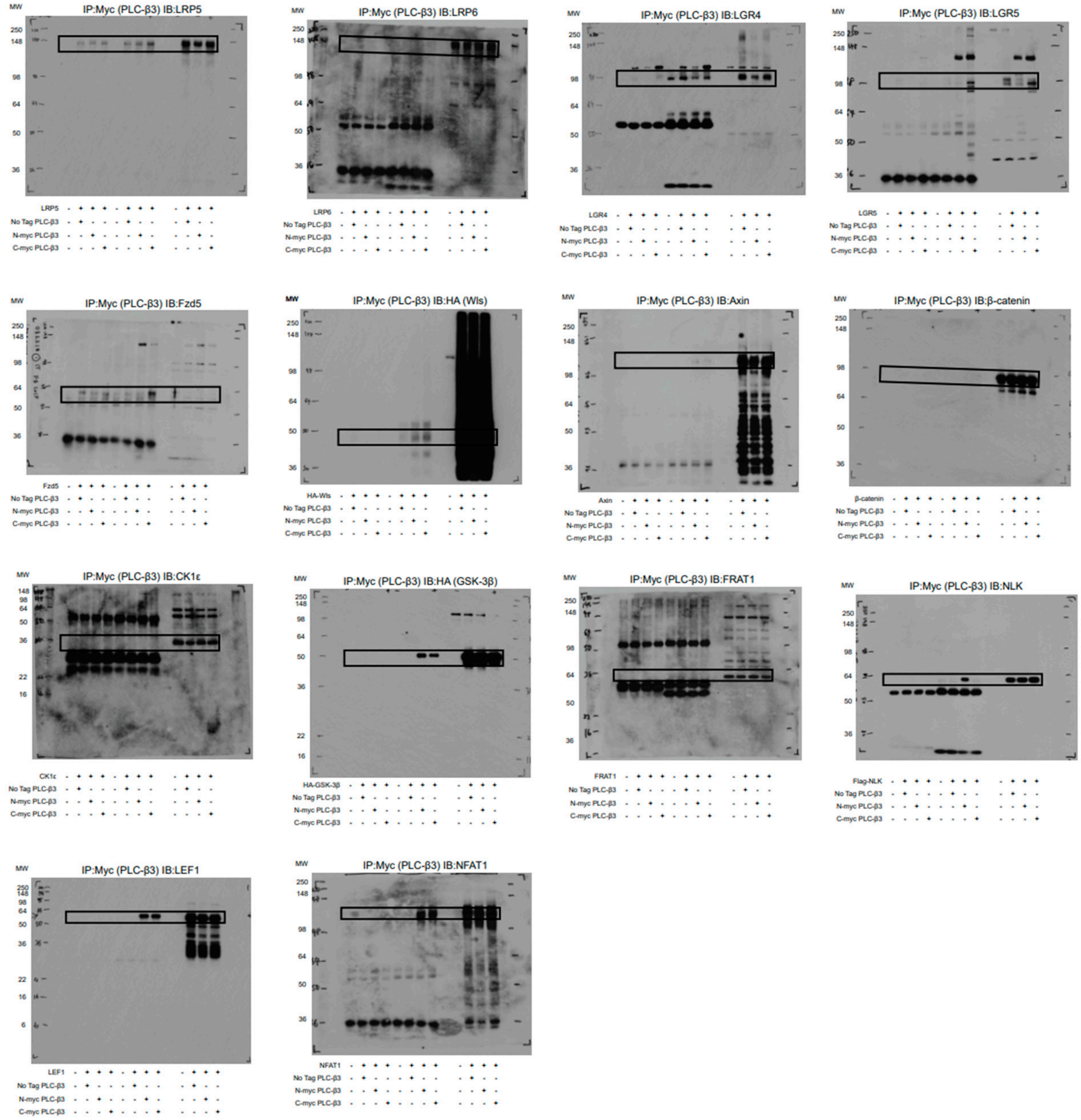

# Original scanned images for Figure 4I

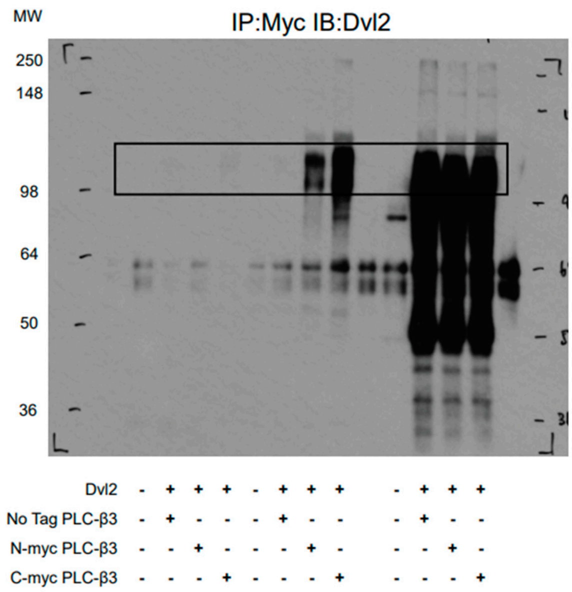


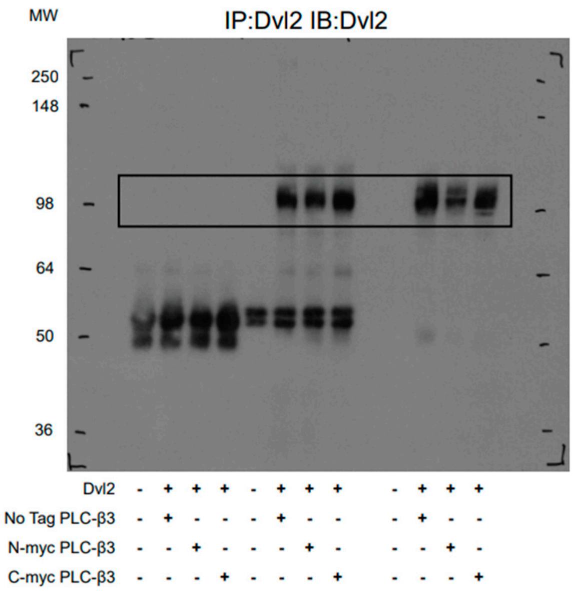


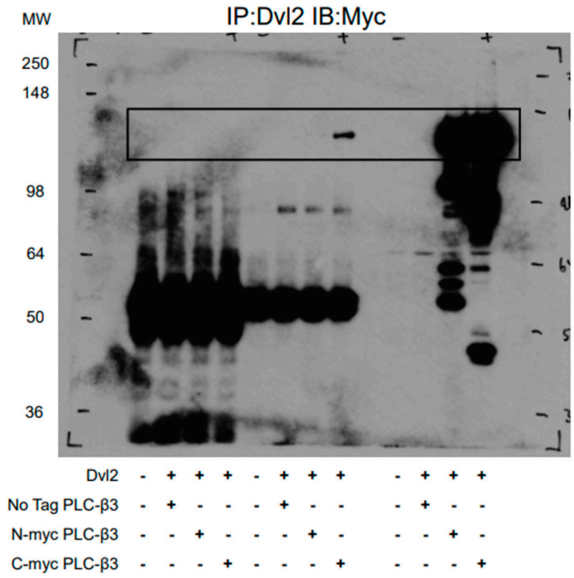


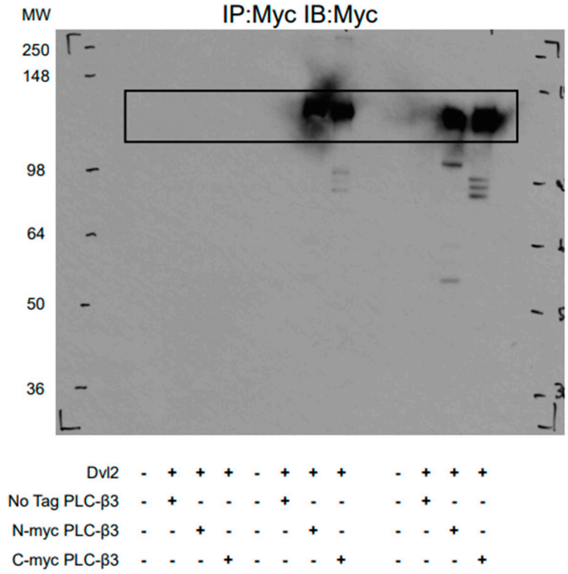

# Original scanned images for Figure 4J

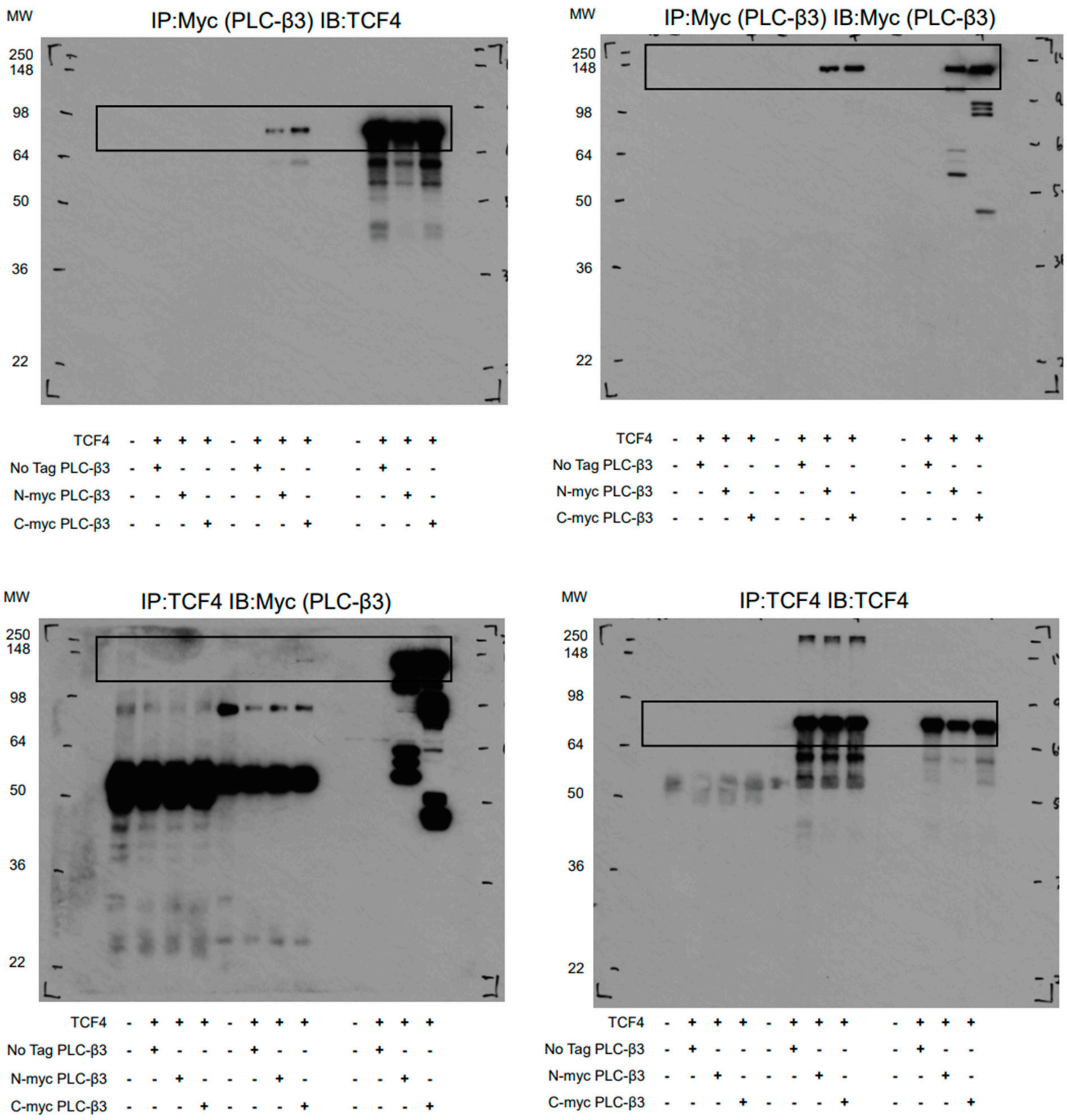

## Original scanned images for Figure 4M

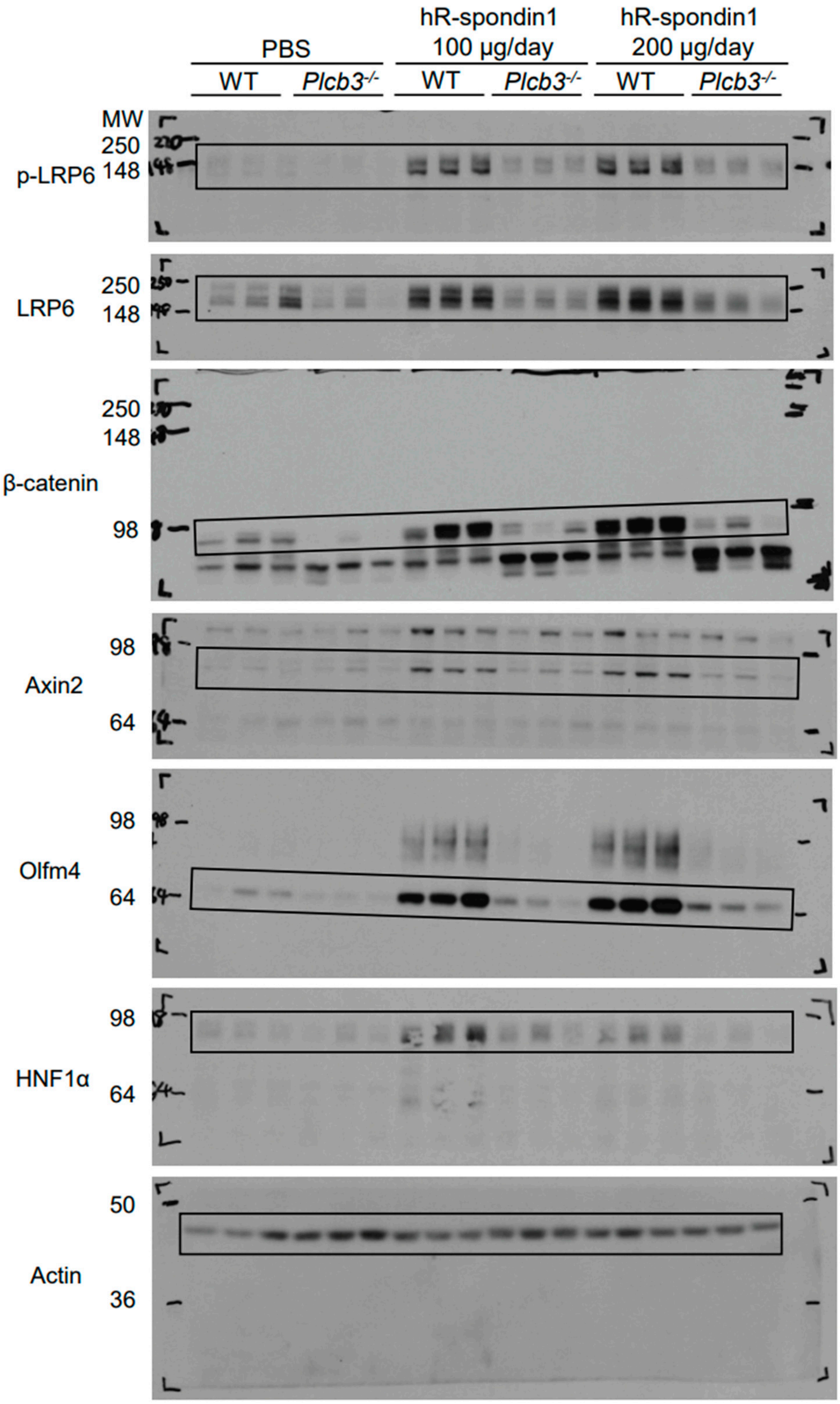

# Original scanned images for Figure S2C

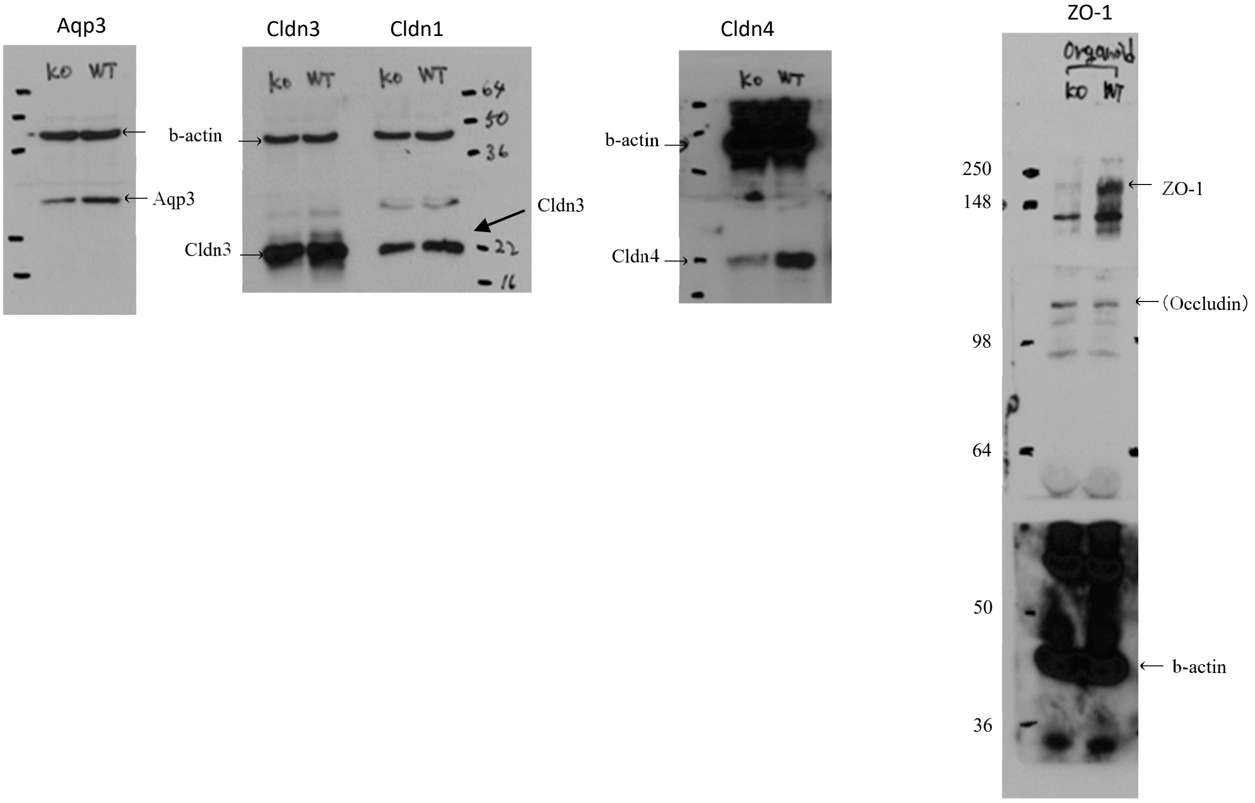

**Original scanned images for Figure S5C**

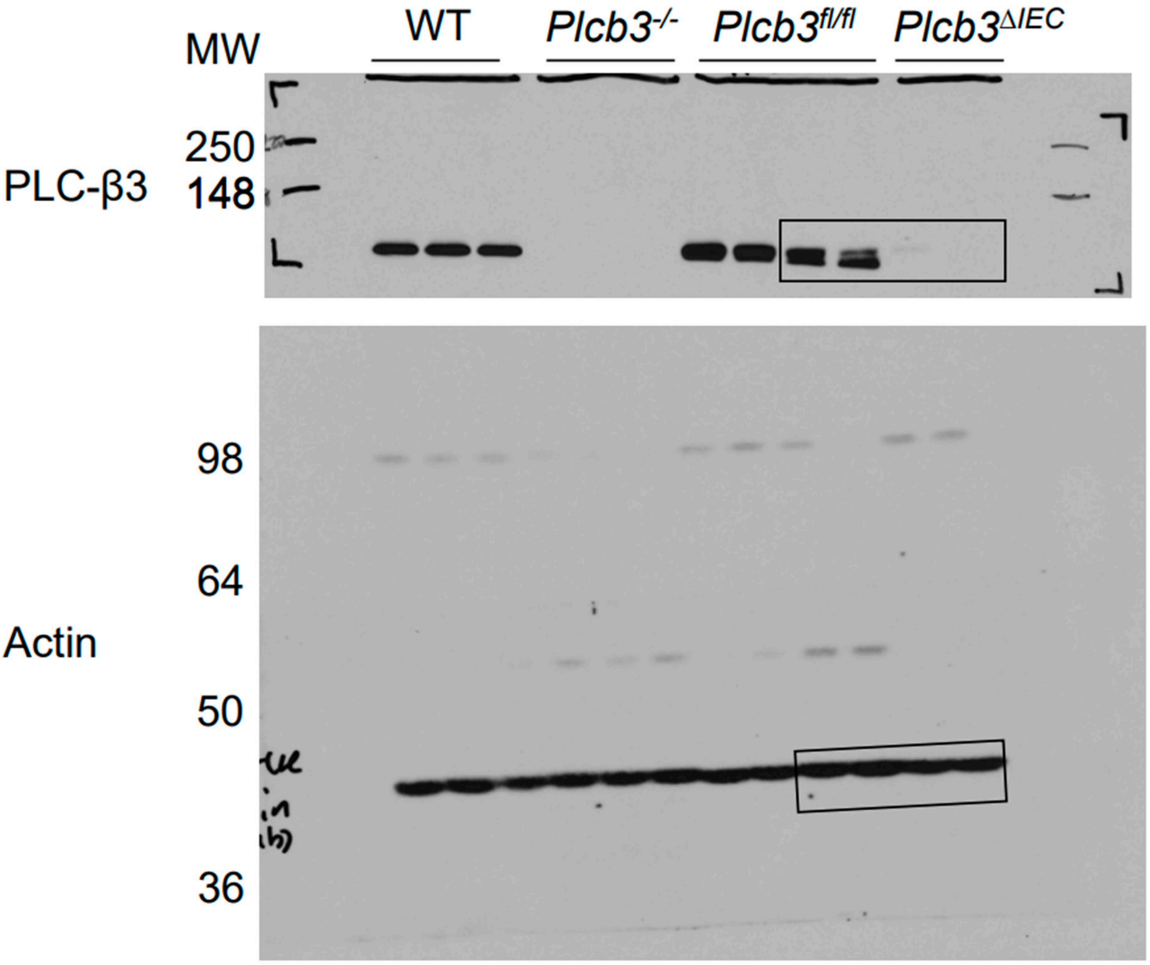

# Original scanned images for Figure S8A

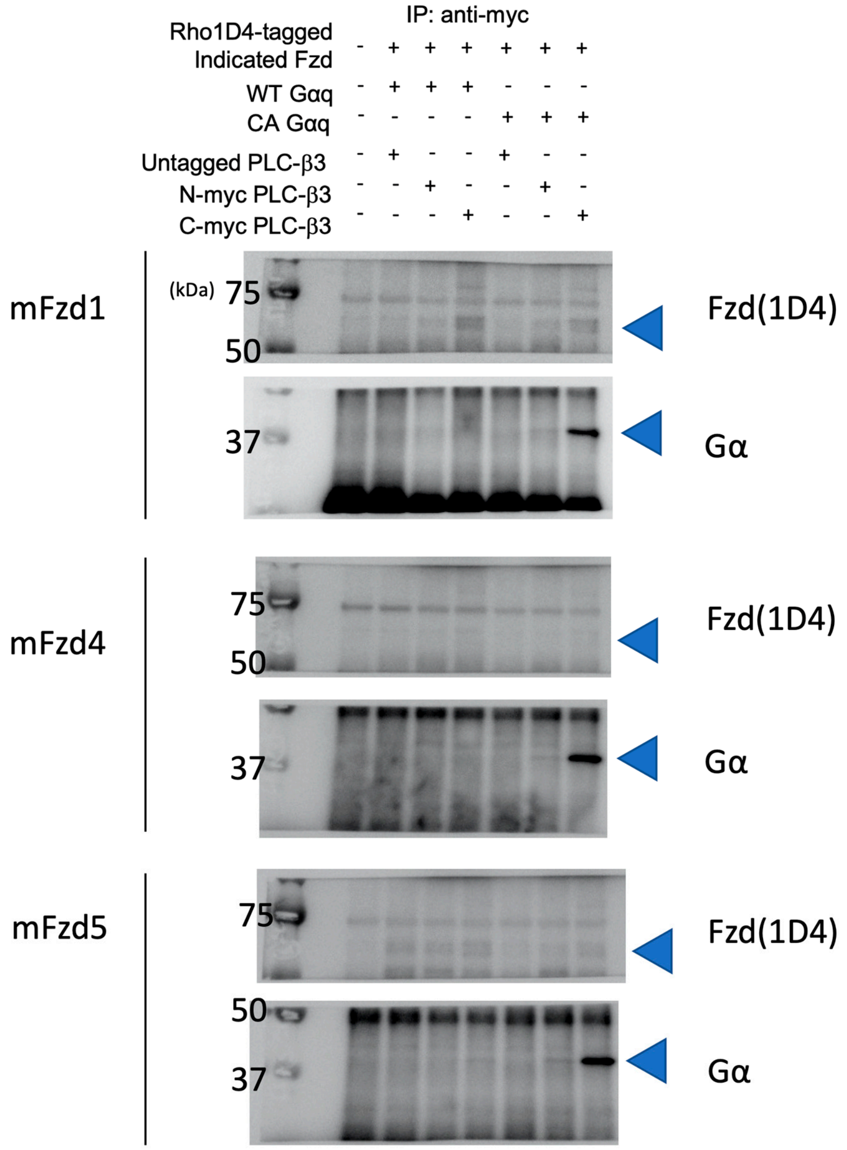

**Original scanned images for Figure S8B**

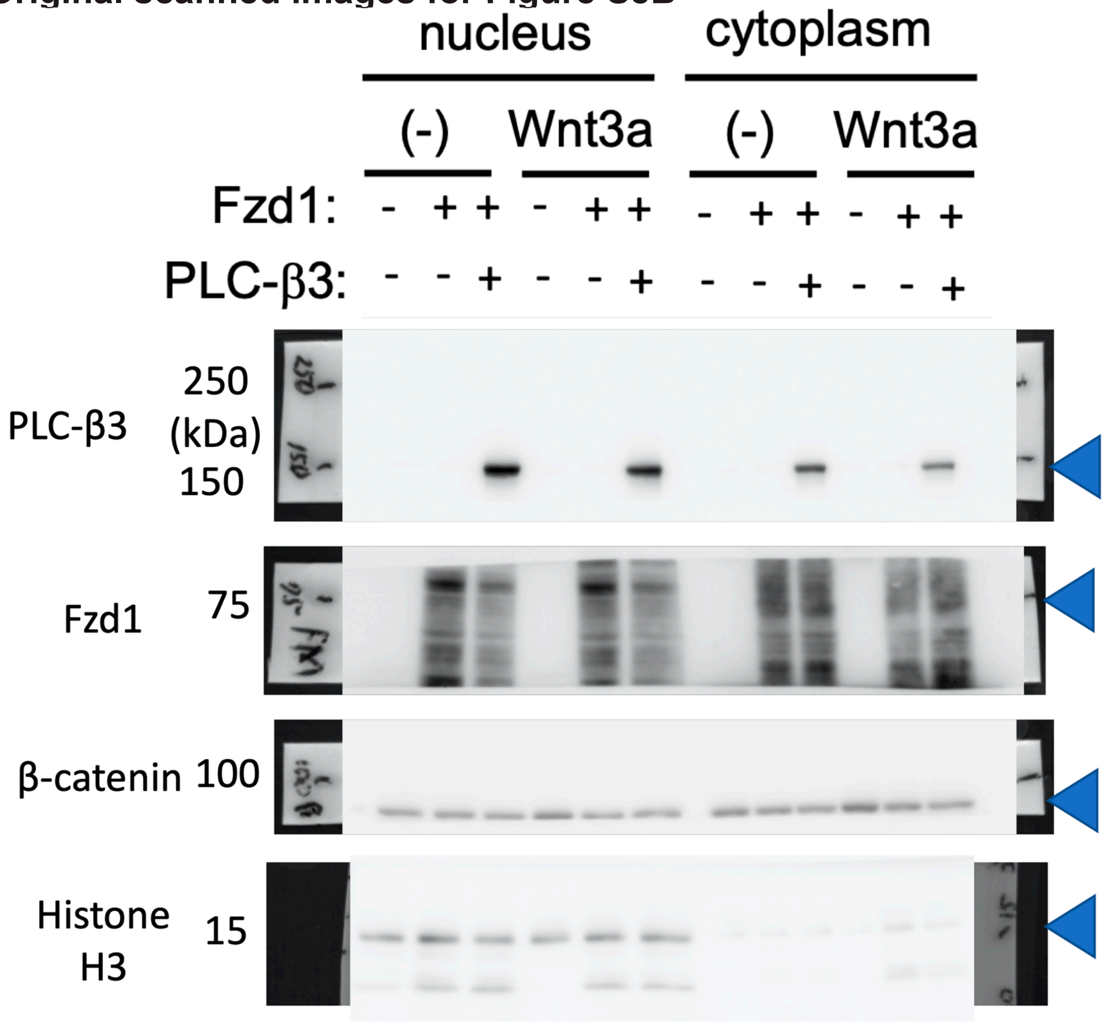

# Original scanned images for Figure S8C

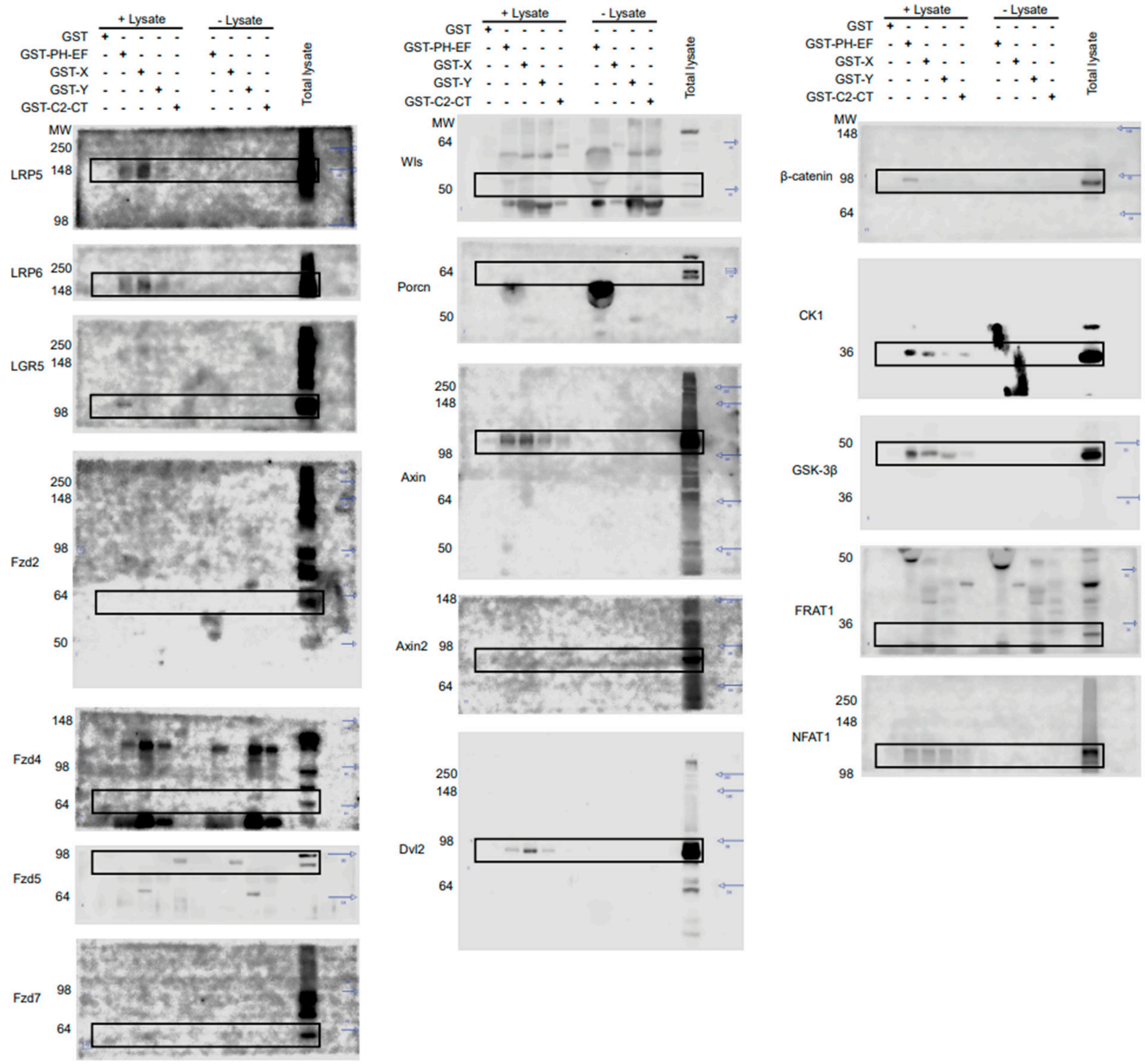

**Video 1. 3D-reconstituted images of ileal crypts from WT mice treated with DSS for 2 days stained by Hoechst (Blue, nuclei), anti-Olfm4 (Green, ISCs), and phalloidin (Magenta, F-actin).**

**Video 2. 3D-reconstituted images of ileal crypts from *Plcb3*$^{-/-}$ mice treated with DSS for 2 days stained by Hoechst (Blue, nuclei), anti-Olfm4 (Green, ISCs), and phalloidin (Magenta, F-actin).**

**Video 3. 3D-reconstituted images of ileal crypts from WT mice treated with DSS for 2 days stained by Hoechst (Blue, nuclei), anti-Ki67 (Green, proliferating cells), and phalloidin (Magenta, F-actin).**

**Video 4. 3D-reconstituted images of ileal crypts from *Plcb3*$^{-/-}$ mice treated with DSS for 2 days stained by Hoechst (Blue, nuclei), anti-Ki67 (Green, proliferating cells), and phalloidin (Magenta, F-actin).**

**Video 5. 3D-reconstituted images of ileal crypts from WT mice treated with DSS for 2 days stained by Hoechst (Blue, nuclei), UEA-1 (Green, Paneth cells), and phalloidin (Magenta, F-actin).**

**Video 6. 3D-reconstituted images of ileal crypts from *Plcb3*$^{-/-}$ mice treated with DSS for 2 days stained by Hoechst (Blue: nuclei), UEA-1 (Green: Paneth cells), and phalloidin (Magenta: F-actin).**

1. R. Elmentaite *et al.*, Single-Cell Sequencing of Developing Human Gut Reveals Transcriptional Links to Childhood Crohn's Disease. *Dev Cell* **55**, 771-783 e775 (2020).
2. H. Xu *et al.*, Transcriptional Atlas of Intestinal Immune Cells Reveals that Neuropeptide alpha-CGRP Modulates Group 2 Innate Lymphoid Cell Responses. *Immunity* **51**, 696-708 e699 (2019).
3. A. L. Haber *et al.*, A single-cell survey of the small intestinal epithelium. *Nature* **551**, 333-339 (2017).
